# Synthesis and characterization of *Curcuma Caesia* plant root extract-mediated ZnO nanoparticles: efficacy as soil conditioner and plant growth promoter

**DOI:** 10.1038/s41598-026-41196-w

**Published:** 2026-04-22

**Authors:** Amritanshu Pathak, Priyvart Choudhary, Garima Kumari, Vinay Kumar Pandey, Shailendra Thapliyal, Ayaz Mukarram Shaikh, Béla Kovács

**Affiliations:** 1 Department of Biotechnology, Ch. Charan Singh University, Meerut, Uttar Pradesh India; 2 Research and Development Cell, Biotechnology Department, Manav Rachna International Institute of Research and Studies (Deemed to Be University), Haryana, 121004 India; 3 Department of Molecular Biology and Biotechnology, Dr. Yashwant Singh Parmar University of Horticulture and Forestry, Solan, 173230 Himachal Pradesh India; 4 Uttaranchal Institute of Technology, Uttaranchal University, Dehradun, 248007 Uttarakhand India; 5 Faculty of Agriculture, Food Science & Environmental Management, Institute of Food Science, University of Debrecen, Böszörményiút 138, Debrecen, 4032 Hungary; 6 Department of Microbiology, Graphic Era (Deemed to be University), Dehradun, Uttarakhand India 248002

**Keywords:** *Curcuma caesia*, *Abelmoschus esculentus*, ZnO nanoparticles, Crop yields, Metabolomics, Biochemistry, Biological techniques, Biotechnology, Chemistry, Plant sciences

## Abstract

Green synthesis of metal oxide nanoparticles is emerging as potential application for sustainable farming. Zinc oxide nanoparticles (ZnO NPs) were successfully biosynthesized by *Curcuma caessia* rhizome extract as a reducing and stabilizing agents in the present investigation and they were applied to test their role as a soil conditioner and growth promoter for *Abelmoschus esculentus*. Characterization of the synthesized ZnO NPs was carried out by UV–Vis spectroscopy, FTIR, XRD, SEM, AFM, dynamic light scattering and ζ-potential analysis. The UV–Vis absorption spectrum exhibited an absorbance at 340 nm, indicating the formation of ZnO and the crystalline nature of this product was confirmed by XRD measurements with average size along (002) plane equals 43 nm. The hydrodynamic size of the nanoparticles was shown to be 217 nm with a ζ-potential of −22.1 mV, reflecting an acceptable colloidal stability. Field and germination studies revealed that seed germination, vegetative growth, flowering, and yield parameters were significantly improved in ZnO NP-treated plants compared to control ones in a dose-dependent manner. Additionally, ^1H-NMR metabolomics of okra pods indicated changes in primary and secondary metabolite profiles, indicating possible modulation of metabolic pathways by ZnO NP. The findings of the study revealed that *Curcuma caesia* – synthesized ZnO NPs acted as an eco-friendly nanomaterial to modulate plant growth and metabolic performance suggesting its possible use toward sustainable farming.

**Supplementary Information:**

The online version contains supplementary material available at 10.1038/s41598-026-41196-w.

## Introduction

Nanotechnology is an emergent multidisciplinary approach that involves the production of molecules in the range of nanoscale size. It is also regarded as new and rapidly developing discipline that involves the formation, processing and use of structures, devices, and systems by manipulating shape and size on a nanometer scale. Nanotechnology term is derived from Greek word nano that means dwarf. Nanoparticles are described as distinct entities with diameter of 100nm or less to 1nm^1^. The biogenic manufacture of nanoparticles also makes use of plant leaf, seed, root, stem, and latex extracts, which serve as reducing or stabilizing agents^2,3^. The numerous applications of nanoparticles in the fields of agriculture, cosmetics, medicine, industries etc. can be attributed to their unique characteristics, which embrace their smaller in size between 1-100nm range, and it has greater surface area to volume ratio, quantum size properties, reactivity, physical strength, and electrical, magnetic, or optical properties. Nano biosensors, nutrition management, nano-pesticides, plant growth regulators, plant growth promoters, and defense against phytopathogens are just a few of the many uses for nanoparticles in agricultures^4^^,^^5^.

Nanotechnology’s use in agriculture has gained global attention in the 21^st^ century due to its impact an all-agriculture system. Researchers discovered that nanotechnology in agriculture could reduce the usage of water, fertilizers, and pesticides, while also increasing agriculture productivity. Indeed, nanotechnology has the potential to play a significant role in the development of sustainable agriculture and precision farming^6,7^. Recent developments in the production of nanomaterials in wide range of sizes and shapes have resulted in variety of applications in the field of medical, environmental sciences, agriculture, and food processing^8^. Through uptake and accumulation nanoparticles have a substantial interaction with higher plants, influencing their transformation and mobility in both terrestrial and aquatic habitats. For humans, animals, and plants to thrive properly^9^. Zinc is a micronutrient that is necessary. Plants suffering from zinc deficit may develop more slowly and produce less. Comparing ZnO NPs to soluble zinc, more *Arabidopsis* growth is inhibited. Similar results have been recorded from lucerne when crop plants treated with ZnO NPs, their seed germination rate is blocked, and their seedling growth is inhibited. Zinc oxide compound is amongst the most fascinating and auspicious metallic nanomaterials. In addition to existence a vigorous reducing agent and an extremely active element, zinc has the capability to willingly oxidize and produce zinc oxide, which is useful for the creation of ZnO NPs^10^.

All over the experimentation, plants were fully-fledged to maturity in natural environment with 60-90% moisture contents of the entire soil water-holding capability. When nanoparticles were additional using, especially at greater rates, plant height, spike and grains are also increased. The findings showed that, in comparison to the control, nanoparticles had a beneficial impact on wheat crop photosynthesis process^2,3,11^. This study grasps importance in emerging a sustainable, green path for bio-synthesizing ZnO nanoparticles by utilizing *Curcuma caesia* rhizome extract, eradicating noxious chemicals, and plummeting environmental impact. The possibility encompasses characterization nanoparticle and assessment of their application in enlightening soil quality, microbial activity, and plant development. Its significance fabrications in encouraging eco-friendly nanotechnology applications for heightened agricultural efficiency and enduring soil health management.

## Material & methodology

For the present study *Curcuma caesia* plant was procured from Jadi-Buti Farm (*Latitude: 30° 20’ 4.4952". Longitude: 78° 3' 13.6116*") place located in Kolhupani, Dehradun, Uttarakhand, India. The plants were sustained in laboratory of the Department of Biotechnology, CCS University, Meerut, Uttar Pradesh, India. The all chemicals used in this study was analytical grade. *Abelmoschus esculantus* (L.) seeds (Pusa Bhindi-5) was purchased from local market.

## Permission

Raw plant materials were collected, and plant species were identified with the AI-based recognition application Flora Incognita. No specific permissions or licenses were required to select *Curcuma Caesia.*

### *Curcuma caesia* rhizome extract preparation

The *Curcuma caesia* rhizomes were dried in the shade after being carefully cleaned to get rid of dust and other contaminants. 50g sample of dried rhizome was weighed, ground into a powder, and then mixed with 100ml of distilled water to dissolved it. The mixture was allowed to settle overnight. After that, the sample was heated to between 70℃ to 80℃ for three hours on a hotplate. A rotary evaporator was used to filter and concentrate the solution, yielding 2% of the residue’s dry weight. A proportion of the extracted mass divided by the mass of crushed rhizome was used to compute and express the express the extraction yield (%, w/w). The rigorous extract of *Curcuma caesia* rhizomes was used for further analytical analysis and also utilization for bio-synthesis of ZnO NPs.

### Bio-synthesis of ZnO NPs

ZnO NPs synthesis was adopted from Rehana et al.,^12^. The bio-synthesis of ZnO NPs utilizing 2 mM Zn (NO_3_)_2_.6H_2_O as the predecessor, at a reaction temperature (R_t_) of 50°C and calcination temperature (C_t_) set on 150°C for 120 minutes, was selected grounded on fundamental physicochemical deliberations to ensure precise nucleation, uniform particle development, and high surface steadiness. The preferred 2 mM precursor concentration make available an optimum steadiness between ion availability and supersaturation, leading to measured nucleation and development of NPs with condensed agglomeration. At this concentration, the reaction takings with appropriate Zn^2+^ ion obtainability to form well-defined nuclei, although avoiding unnecessary particle coalescence or heterogeneous size circulation that every so often materializes at higher molarities. The R_t_ improves the kinetics of hydrolysis process as well as condensation reactions by cumulative ion flexibility and solubility, thereby encouraging homogeneous nucleation and better crystallinity. This reasonable temperature confirms adequate reaction stimulation without triggering the putrefaction of organic capping or reducing agents, sustaining the integrity of efficient surface groups. The calcination step is critical for the elimination of residual organic composites, water particles, and nitrate species, simplifying the comprehensive conversion of Zn (OH)_2_ arbitrates into transparent ZnO. Protracted space heating at this medium temperature allows steady dehydration and structural restructuring, enlightening the crystallinity of nanoparticles while thwarting excessive grain growth or sintering, thus preservative the nanoscale syllable structure and high surface area looked-for for catalytic and biomedical applications.

**Fig. 1:**
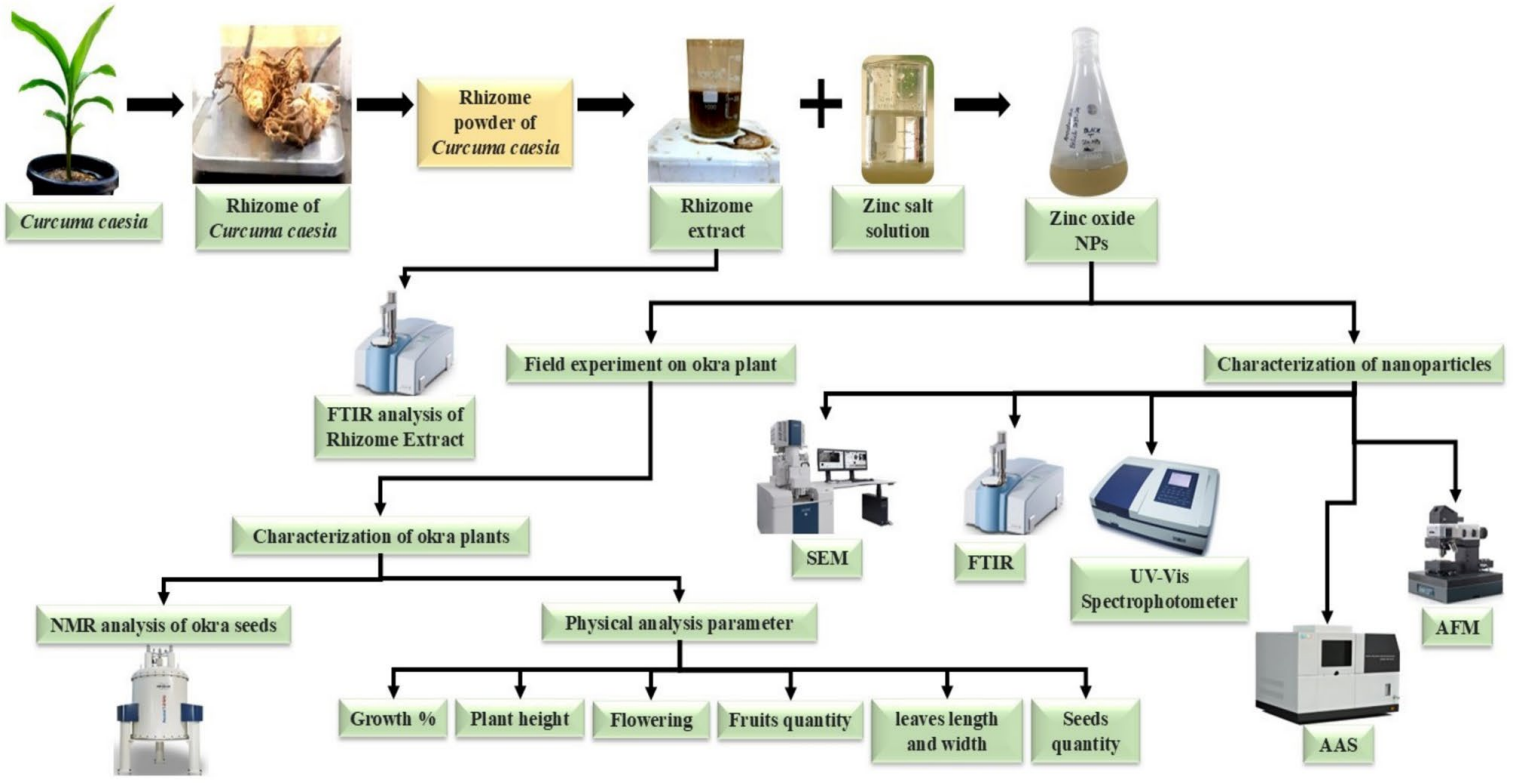
Graphical representation of synthesis and characterization analysis of *Curcuma caesia* rhizome extract and Bio-synthesized ZnO NPs.

50mL of 2mM zinc nitrate hexahydrate Zn (NO_3_)_2_.6H_2_O was added drop wise to 100 mL of *Curcuma caesia* rhizome extract under mechanical stirring and heated at 50°C for 90min. After 90min the solution temperature maintains at 37°C for 120min at mechanical stirring. During the reaction color changes confirmed the formation of zinc hydroxide complexes (Figure 1). The dark brown precipitate that is present in bottom of beaker was later separated by centrifugation process at 15000 RPM for 15min and washed quite a few times with distilled water until the pH of the suspension reached ~7. The solid product was transferred to a ceramic crucible, dried methodically, and calcined at 150°C for 120min, then it stored for further analysis purpose.

### Characterization of *Curcuma caesia* rhizome extract and bio-synthesized ZnO NPs

The *Curcuma caesia* plant rhizome extract and ZnO NPs were subjected to FTIR analysis was performed by the instrument Nicolet Summit LITE” (Instrument Serial No.: BFJ2010008). FTIR analysis conducted for check the functional groups were present in the *Curcuma caesia* plant rhizome extract and ZnO NPs^13^. The UV absorbance of the synthesized ZnO nanoparticles was measured in UV-Vis single beam spectrophotometer (Model no. LI-295) operated at a spectral bandwidth resolution of 1 nm optical path at ambient temperature^14^. The phase configuration and crystallinity of the ZnO nanoparticles was observed using X-Ray Diffraction technique (Model- Rigaku Smart lab X-ray diffractometer using filter 1-D Cu Kβ radiation). Morphology and rudimentary mapping of sample was considered by scanning electron microscope (SEM) and its model (Carl Zeiss EVO 18)^13^^,^^15^. Topography of the nanoparticles was categorized by using Atomic Force Microscopy (AFM) (Model: A100,A.P.E. Research, Italy). ^1^H-NMR spectrum of *Abelmoschus esculantus* (L.) seeds extract was executed by using JEOL Resonance JNM-ECS400 spectrometer at 400MHz with 5mm inverse probe (Figure 1).

The electrostatic potential that arises at the shear plane of a nanoparticle, which is of concern to both the surface charge and the local intermediate of the particle is known as ζ-potential. The ζ-potential and size were analyzed by Zeta sizer Ver. 7.12 (Malvern Panalytical; Instrument Serial No.: MAL1171112). Utilizing stage investigation light scattering mode and maintain appropriate room temperature for recording all the measurements. For the calculation of the ζ-potential, in this the Smoluchowski equation that is mentioned below^16^.

$$\nu = \left(\frac{\varepsilon E}{\eta }\right)\upzeta$$

whereas, ν = electrophoretic velocity, η = viscosity, ε = electrical permittivity of the electrolytic solution, and E = electric field.

### Field Experimental design

**Fig. 2:**
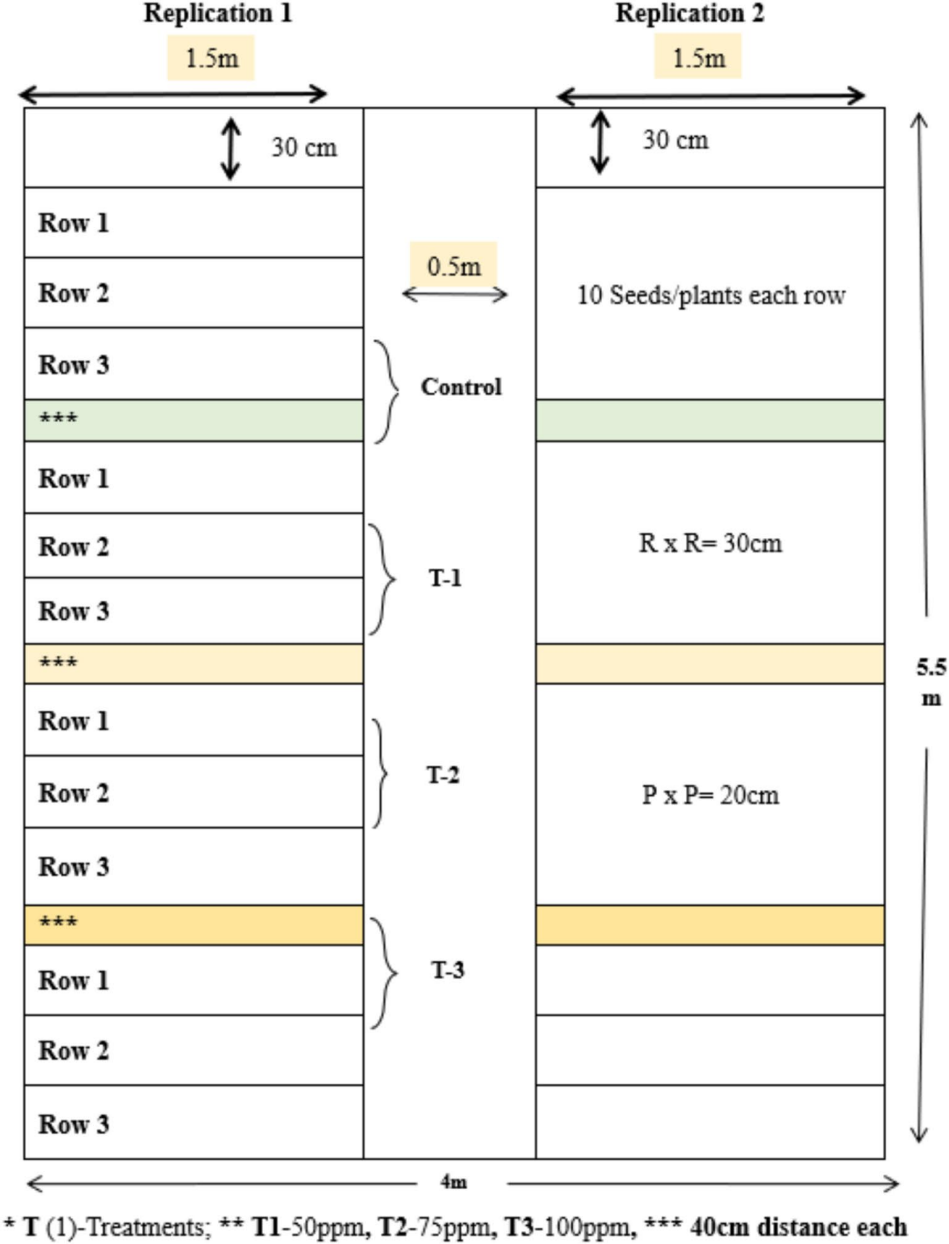
Graphical representation of field experimental design.

The study rummage-sale a randomized comprehensive block design that was set up as a split plot with two replications in the field, with three rows each of five concentrations of treatments as well as control (Figure 2). Complete experimental plots were measured into 5.5 x 4 meter and divided into two equal parts having each 1.5 meters plot size and gap between them by 0.5 meter. Both plots further divided as per concentration of NPs treatment (such as T1, T2, T3, T4, T5 & control) having further three rows of replication of each treatment (such as R1, R2 and R3). Distance between each row having 30 cm. Distance between two treatment plots (T1 and T2) having 40 cm. Each treatment having three rows of replication such as (T1 having R1, R2 and R3 replications) respectively. Numbers of plants in each rows having 10 (means Row 1 having 10 plants of okra and respectively). So, number of plants per treatment having 30 such as (R1=10 plants; R2=10 plants and R3=10 plants). Total numbers of plants utilised for analysis of morphological traits was 180. Soil texture diverse, with the 0-10 cm layer exhibiting a stable composition. The pH levels ranged from 6.23 to 6.43, that is indicating a trend towards neutral conditions. Nutrient profile or organic matter gratified varied, with the 0-15 cm layer screening stability at 1.9-2.8%, which also depends on the weather condition.

#### Seed treatment and germination experiments

In directive to study the consequence of different concentration of ZnO NPs on germination of *Abelmoschus esculantus* (L.) seeds a comprehensive randomized design with 3 replication was employed. The experimental treatments contained within three concentrations that’s 50ppm, 75ppm and 100ppm of ZnO NPs and also a control (without ZnO NPs). The seeds of uniform size were selected and drenched in nanoparticles suspension and in distilled water (control) for 120min. After 120min of imbibition the seed leachates were then transferred to the petri plate that is contain wet filter papers and as three groups of 10 seeds in each petri dish. The seeds were well thought out to be germinated when radical accomplished a length of 1mm and plumule had just unfolded.

#### Assessment of morphological and biochemical response

The swallowed seeds (10seed/plot), have three replicates of each concentration were transferred to experimental field to study the morphological and biochemical response. The field were irrigated with the nanoparticle’s solution of respective concentration and a control with distilled water on intermission of 10 days. After 60 days of growing of plants were uprooted for morphometric analysis such as measurement of germination percentage, plant height, leaf length, leaf width, flowering percentage pod/crops and pod weight as a morphological response. In order to study the metabolites responses, pod extract was used in DMSO solvent for NMR analysis.

### Statistical analysis

All the experimentations were accomplished in triplicate manner and the results are expressed as mean with statistical error. To test for differences, the LSD with Duncan’s post hoc test, as well as on-way analysis of variance, were employed with IMD SPSS ver. 16.0 software at as significance level of 0.05%.

## Result and discussion

ZnO NPs treatment completely prejudiced seed germination, vegetative development, and yield characteristics, dependable with preceding conclusions reporting nanoparticle mediated enhancement in crop make-up and efficiency. Supplementary Figure 1(a-h) demonstrates the broad-minded phases of Abelmoschus esculentus cultivation under ZnO NPs treatment. Supplementary Figure 1(a) demonstrations the fortified field before sowing, while Supplementary Figure 1(B) demonstrate NP treated okra seeds, signifying seed priming that augments propagation potential concluded enhanced enzymatic movement and membrane constancy. The sprouted seedlings show in Supplementary Figure 1(c) and early vegetative stage showing in Supplementary Figure 1(d) reveal improved vigor and chlorophyll pigmentation in NPs treated plants, portentous heightened nutrient acceptance and photosynthetic effectiveness. The flowering phase showing in Supplementary Figure 1(e) which appeared earlier in NPs treated plants, prospective due to amplified auxin and cytokinin directive encouraged by ZnO NPs. Pod commencement Supplementary Figure 1(f-g) and maturity show in Supplementary Figure 1(h) that looks alike healthier and larger fruit development associated to control plants, confirmative the application of Zn as a micronutrient stimulating simple carbohydrate metabolism and multiplicative growth.

### Characterization of *Curcuma caesia* rhizome extract

#### FTIR analysis of *Curcuma caesia* rhizome extract

**Fig. 3:**
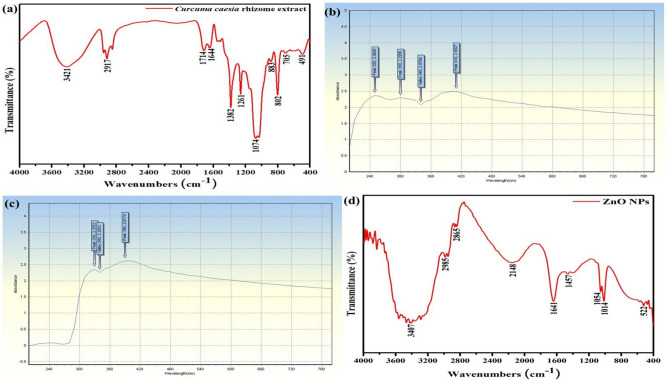
(**a**) FTIR analysis of *Curcuma caesia* rhizome extract; (**b**, **c**) UV-Vis Spectrophotometer of *Curcuma caesia* rhizome extract mediated ZnO NPs; (**d**) FTIR analysis of *Curcuma caesia* rhizome extract mediated ZnO NPs.

The investigation of the FTIR spectrum of the rhizome extract of *Curcuma caesia* showing characteristic peaks of organic compounds of different functional groups in Figure 3 (a), that’s including hydroxyl compound at 3421cm^-1^, O-H stretching at 2917cm^-1^, C=C cyclic ring at 1714cm^-1^, amide-I and carboxylic acids at 1644cm^-1^, C-H alkane band at 1382cm^-1^, aromatic C-O stretching at 1261cm^-1^, C-O stretching at 1074cm^-1^, aliphatic amines at 883cm^-1^, alkene at 802cm^-1^ and 705cm^-1^ and halogen compound at 491cm^-1^^17–21^. The existence of comparable groups likes O-H, C=C, N-H, C-H and C-O has been found in the ethanolic extract of the *Curcuma caesia* rhizome extract^22^. Turmeric species contains a variety of phytocompounds that are comparable to those found in their rhizomes and may have therapeutic and economic applications^23^. Similarly, a study reported by Bhattacharya et al.,^24^ the importance of FTIR approach for spectrum analysis of curcuma species.

### Characterization of *Curcuma caesia* rhizome extract mediated ZnO NPs

#### UV-Vis spectrophotometer of nanoparticles

The peak range for ZnO NPs in UV-Vis spectroscopy lies between 300nm - 360nm. The peaks corresponding to ZnO NPs started appearing at 0 hours observations in Figure 3 (b). After 24 hours, the absorption peaks of ZnO NPs were recorded at 340 nm in Figure 3 (c)^25^. UV-Vis spectroscopy is a broadly castoff technique for the primary characterization of various synthesized nanoparticles due to its easy, fast, simple, sensitive, and selective nature. This is because nanoparticles have exceptional optical properties that are sensitive to their size, shape, agglomeration state, concentration, and refractive index that is close to their surface^26^.

#### FTIR analysis of *Curcuma caesia* rhizome extract mediated ZnO NPs

FTIR analysis spectrum of the *Curcuma caesia* rhizome extract mediated ZnO NPs showed characteristic peaks of organic compounds of different functional groups in Figure 3 (d), that’s including hydroxyl compound at 3407cm^-1^, C-H stretching at 2985cm^-1^, alkane group at 2865cm^-1^, C=N stretching at 2148cm^-1^, O-H banding at 1641cm^-1^, alkyne CH group at 1457cm^-1^, 1054cm^-1^ assigned to deformation mode of the CH_3_ group indicate the occurrence of some very small residues of zinc precursor-zinc acetate, C-O stretching at 1014cm^-1^. In addition, a small peak appears at 522cm^-1^ which is associated with the characteristic stretching vibration mode of Zn-O bond^27–31^.

#### XRD analysis of *Curcuma caesia* rhizome extract mediated ZnO NPs

**Fig. 4:**
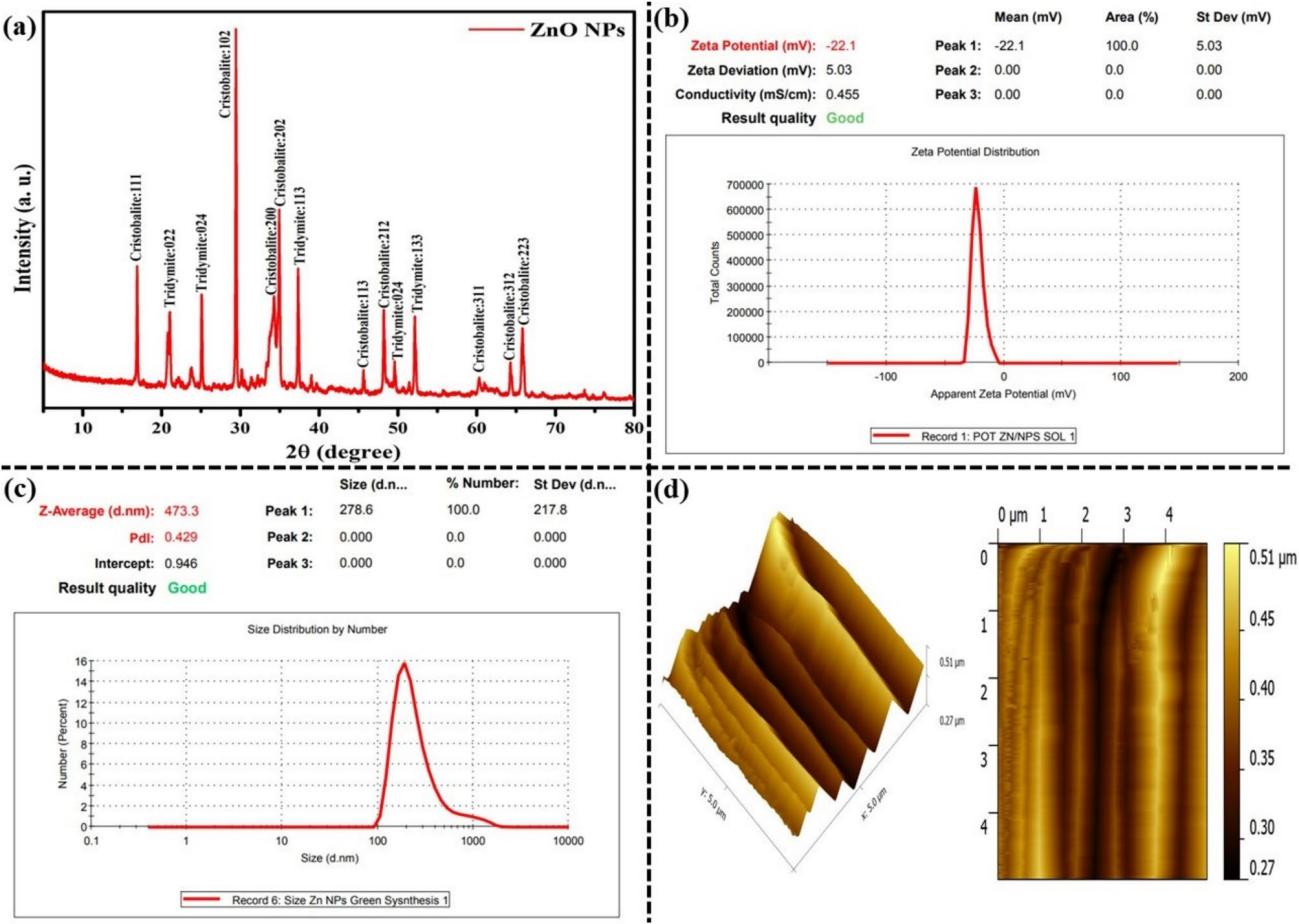
(**a**) XRD analysis of *Curcuma caesia* rhizome extract mediated ZnO NPs; (**b**, **c**) ζ-potential and size analysis of *Curcuma caesia* rhizome extract mediated ZnO NPs; (d) AFM analysis of *Curcuma caesia* rhizome extract mediated ZnO NPs.

XRD pattern of *Curcuma caesia* rhizome extract mediated ZnO NPs measured at 2°/min scanning rate to determine their purity, crystal structure, crystalline phase, and average crystal size. The results of ZnO NPs analysis are shown in Figure 4 (a). The results are confirmed that the prepared ZnO NPs have the crystalline nature. Peaks were detected along with Braggs reflection planes or miller indices of (111) Cristobalite, (022, 024) Tridymite, (102, 200, 202) Cristobalite, (113) Tridymite, (113, 212) Cristobalite, (024, 133) Tridymite and (311, 312, 223) Cristobalite respectively. Which are placed at 16°, 21°, 25°, 29°, 34°, 35°, 37°, 46°, 48°, 49°, 52°, 61°, 64° and 66° respectively, correspond to the angle of diffraction (h). The main XRD speak of the sample fits the crystallo-graphic planes (111) at 2θ = 16 that corresponding to the anatase category, highlighting the fact that anatase is the main crystalline phase of as-prepared ZnO NPs. The miller indices (h k l) values are well fitted with the standard JCPDS card number: 00-036-145^32^. In this study, Debye Scherrer formulary methodology was used to determine the particle size measurement by using the following formula: d = 0.94 λ/β Cos θ, where d is the average diameter of the ZnO NPs, wavelength (λ) is the X-ray radiation source and diffraction angle (θ) corresponding to the XRD peak of angular full width at half maximum (FWHM) are all put in the formula Average crystallite size (assumed β = 0.20° for all peaks): ≈ 43.01 nm and Range: ≈ 40.11 nm (smallest) to 47.36 nm (largest)^33^. It was observed that the ZnO NPs crystal was generally among 15-20 nm. However, the crystalline nature and average crystalline size values are identical near match with previously observed results^34^. Moreover, a minor variation in phase shift, peak strength and average crystalline size occurred due to the synthesis method, herein organo sulfur and poly-phenolic compounds in said extract offered the green ZnO NPs stronger and crystalline peaks.

#### ζ-potential and size analysis of *Curcuma caesia* rhizome extract mediated ZnO NPs

The ζ-potential and size distribution of *Curcuma caesia* rhizome extract mediated ZnO NPs were examined by using the dynamic light scattering (DLS) technique. Surface charge on a particle id often measured by the ζ-potential, which also defines the colloidal stability. Suspensions that exhibit 5.03mV are generalized as stable colloids. ζ-potential of the ZnO NPs in distilled water was restrained as 22.1mV, and which indicates that they are significantly anionic. The dispersion capacity of synthesized ZnO NPs is thus confirmed and supported by the ζ-potential analysis. The binding affinity of the extracted compounds with the NPs results in a negative surface charge, stabilizing the ZnO NPs and lowering their tendency to aggregate [Figure 4(b)]. Using dynamic light scattering, the hydrodynamics size of the particles was ascertained, and for the aqueous synthesis of ZnO NPs, it was found that to be 217nm, as illustrated in Figure 4(c). In comparison to the size determined from SEM image, the size distribution graph demonstrates that the particle size is larger and more polydispersed. Due to the technique’s bias towards measuring larger particles, ZnO NPs detected by DLS have an enhanced size^16^. Unalike functional groups such as carbohydrates, polysaccharides, pectin, etc. existing in plant extract that is adsorbed on the surface of the NPs may affect its ζ-potential. The metabolites that are absorbed on the surface ZnO NPs and ζ-potential are closely related^35^.

#### AFM Analysis of *Curcuma caesia* rhizome extract mediated ZnO NPs

AFM analysis gives us understanding about the topography, irregularity of nanoparticles. AFM image that is clearly demonstrate smooth nanoparticle with capping of phytochemicals over the surface of nanoparticles [Figure 4(d)]. The AFM could provide the digital images that showed the quantitative measurements of surface characteristics like surface height, average roughness, and three dimensions. The probe of the microscope associated to a cantilever that is scanned over the comprehensive surface of glass piece, with small repulsive force between glass and probe. The topographical image of nanoparticles showed that their surfaces were uneven and covered in many dents. Surface length was 0.51μm and average square roughness was 0.27μm. The average roughness (Ra), typically lesser than Rq value, it can be appraised by utilizing the empirical relation such as, Ra = 0.8×Rq, and the yielding of Ra = 0.216µm (such as 216 nm) in range. 2D and 3D atomic force microscopy (AFM) imaging of ZnO NPs (A) Topographical 3D picture of ZnO NPs obtained using unfiltered AFM data (B) A synthetic 2D picture^36^.

#### SEM Analysis of *Curcuma caesia* rhizome extract mediated ZnO NPs

SEM analysis was utilized for the study of surface-morphology and distribution of ZnO NPs at different enlargement range. SEM analysis of ZnO NPs at 100x magnification, 200μm range of area depicted particles to be present in gatherings as well as scattered particle of nanomaterials. On enhancing the magnification to 500x, 50μm range of area uniform distribution of particles was detected along with agglutinated singly and spherical form of particles. Similarly, on enhancing the magnification to 1000x magnification, 20μm range of area particles also depicted uniform distribution and agglutinated as well as porous particle. A portion of carefully chosen area was visualized on higher magnification, 1000x, 20μm range of area agglutinated particles, have spherical particles in size and other small particles were also observed disseminated in surface morphology. Particles size was calculated through formula:

$SEM Size analysis \left(nm\right)=\frac{Area}{WD}$** Area and width converted into nm then divided.

**Fig. 5:**
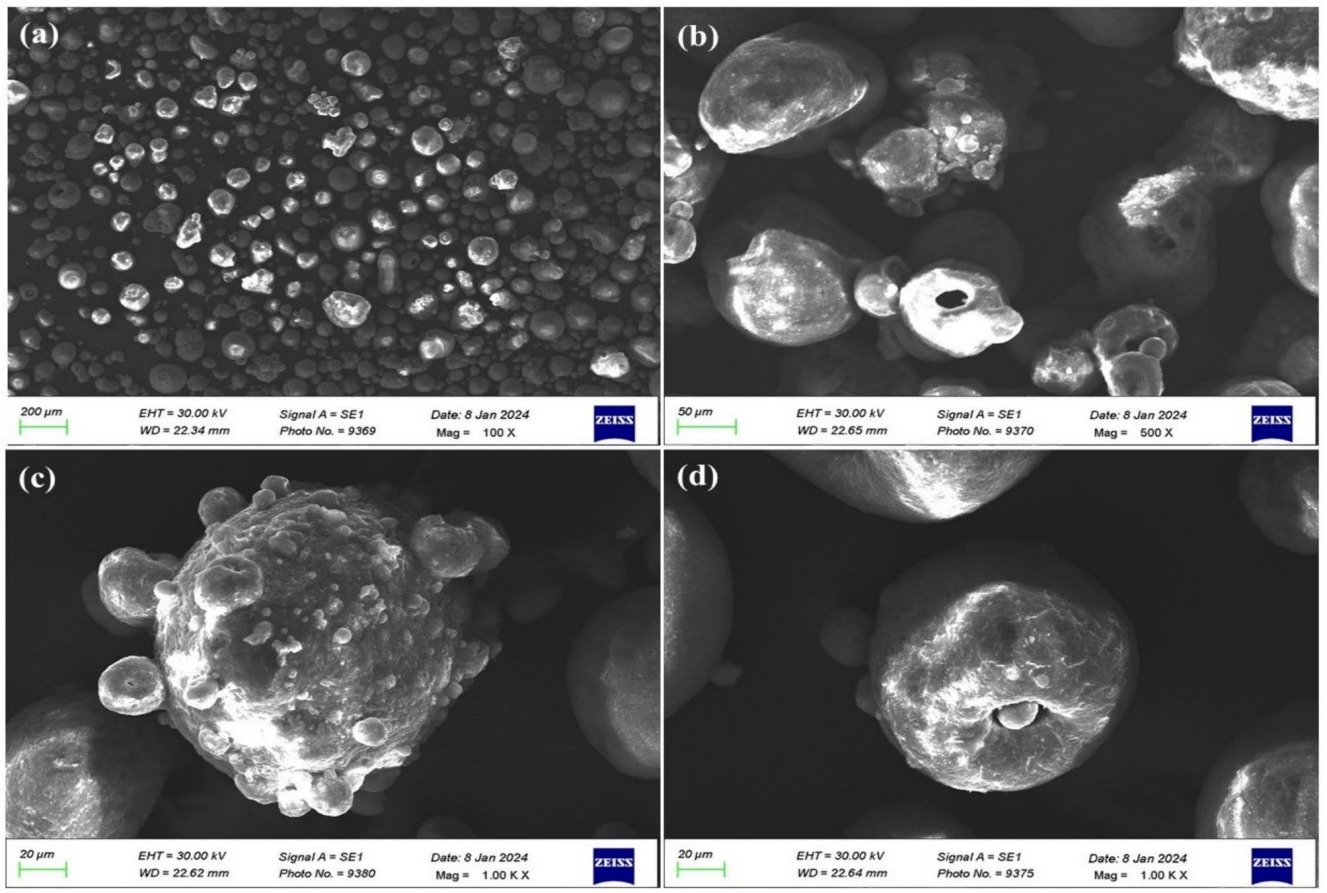
SEM Analysis of *Curcuma caesia* rhizome extract mediated ZnO NPs.

After this calculation the result was obtained in SEM analysis of ZnO NPs is 111.7nm, 453nm, 1132nm and 1131nm show average in size that’s mention in Figure 5 (a, b, c, d) respectively. The divergence between the SEM (size range as 100-1100nm) and DLS (size as 217nm) particle size standards ascends from the diverse dimension principles and the accumulation behaviour of ZnO NPs. SEM make available surface morphological size of dried out and conceivably agglomerative particles, screening both discrete NPs and their larger gatherings formed due to van der Waals holds, hydrogen bonding, or remaining phytochemicals from *Curcuma caesia* extract. Hence, SEM frequently displays a broader size range between (100-1100 nm), on behalf of accumulated assemblies rather than individual crystallites. In dissimilarity, DLS procedures the hydrodynamic diameter of nanoparticles adjourned in solution, which embraces the constituent part core plus any solvation and plugging layers. The average DLS size of 217nm designates that in aqueous scattering, most ZnO NPs exist as smaller, abstemiously steady aggregates moderately than as single large cumulations^37–39^.

### Application of *Curcuma caesia* rhizome extract mediated ZnO NPs in field experiment

*Abelmoschus esculantus* (L.) variety (Pusa Bhindi 5), were selected for the experiment. *Abelmoschus esculantus* (L.) variety was created by the Central Variety Release Committee of the Indian government and released by the ICAR-Indian Agricultural Research Institute, Pusa, New Delhi. *Abelmoschus esculantus* (L.) (Pusa Bhindi-5) was created to increase *Abelmoschus esculantus* (L.) yield and production in North India during the kharif season. For the Kharif season in the northern plains, *Abelmoschus esculantus* (L.) (Pusa Bhindi-5) -(DOV-66) is a high-yielding *Abelmoschus esculantus* (L.) variety with excellent fruit quality and resistance to the Bhindi Yellow Vein Mosaic Virus^40^.

#### Effect of ZnO NPs on seed germination

The consequences revealed that leachate conductivity is more in nanoparticles treated seeds compared to the control seeds. In the propagation was observed in this experiment that’s show the positive effect of ZnO NPs treatment on *Abelmoschus esculantus* (L.) seeds. The percentage of seed germination augmented in all treatments, but the maximum seed germination percentage of 94.6% was observed in 50ppm and lowest of 71% was observed in control (Table 1 and Supplementary Table 1, Table 2, Table 2.1, Table 2.2). This heightened germination may be accredited to the photo-generation of active oxygen such as superoxide and hydroxide anions that improved seed stress confrontation and encouraged for quick germination.

**Table 1:** Effect of ZnO NPs on seed germination the seedling growth of *Abelmoschus esculantus* (L.)

**Concentration (ppm)**	**Germination %**	**Plant height (cm)**	**Leaf height (cm)**	**Leaf Width (cm)**	**Flowering %**	**Pods per crop (mean ± SE)**	**Pod weight (gm) (mean ± SE)**	**Per pod seed quantity**
Control	71.00±0.953	13.26±0.550	6.83±0.305	8.70±0.200	51.60±0.700	4±1.0	10.43±0.375	30±0.608
50	94.63±0.550	19.46±0.503	9.63±0.378	12.20±0.360	71.07±0.975	8±1.0	23.11±4.251	82±0.608
75	92.85±0.501	15.36±0.568	9.40±0.100	10.80±0.264	38.40±0.556	6±1.0	26.96±2.608	97±0.608
100	85.53±9.74	24.44±0.528	12.40±0.458	12.97±0.060	88.40±0.556	16±7.09	24.133±0.321	91±1.15

Mean in each column are significantly by P>0.05 value.

#### Effect of ZnO NPs on plant growth

The effect of ZnO NPs treatment on the plant height was significant impact (Table 1). The plant height increased in treatments 100ppm and 50ppm but decreased in 75ppm and controlled. The highest plant height of 2.44cm was observed in 100ppm followed by 19.46cm in 50ppm and the lowest of 13.26cm in controlled (Supplementary Table 3, Table 3.1, Table 3.2). The leaf length increased in treatments 100ppm, 50ppm and 75ppm as compared to control. The height leaf length of 12.40cm was observed in 100ppm followed by 50ppm have 9.63cm was observed and lowest of 6.83cm in control (Supplementary Table 4, Table 4.1, Table 4.2). In leaf width the highest width was observed of 12.95cm in 100ppm and 12.20cm in 50ppm and lowest of 8.70cm in controlled (Supplementary Table 5, Table 5.1, Table 5.2). The flowering percentage in all treatments highest flowering of 88.40cm was observed in 100ppm and 71.07cm in 50ppm but the lowest flowering of 38.40cm was observed in 70ppm as compared to control (Supplementary Table 6, Table 6.1, Table 6.2). The pod per crops increased in 100ppm concentration was 16 pods and lowest pod obtained in control was 4 pods (Supplementary Table 7, Table 7.1, Table 7.2). In weight of pod of *Abelmoschus esculantus* (L.) slightly change, more weight was observed in 75ppm is 26.96gm and 24.13gm was observed in 100ppm but in control it showed lowest pod weight is 10.43gm (Supplementary Table 8, Table 8.1, Table 8.2). In the seed’s quantity per pod, highest quantity of 97 in 75ppm and lowest of 30 in control was observed (Supplementary Table 9, Table 9.1, Table 9.2). At moderate concentration (75 ppm), the treatment likely optimized the plant reproductive resource allocation able to enhancing fertilization effectiveness, pollen viability as well as nutrient translocation towards development pods. This concentration helps to promoted balanced hormonal signaling such as (auxins, gibberellins, and also cytokinin) leading to better seed development. At higher concentration (100 ppm), the treatment able enthused floral encouraged pathways but hypothetically imposed physiological stress or hormonal imbalance that inadequate successful fertilization or pod development. The response pattern recommends a dose-dependent biphasic effect, where moderate levels enhance reproductive efficiency, while higher levels principally promote flowering but not succeeding seed development.

Similarly, connected to modest concentrations of ZnO NPs have been revealed to improve seed germination, growth of root and shoot, chlorophyll percentage, and antioxidant enzyme activity, however higher concentrations of NPs cause phytotoxic effects^41^. Ramzan et al.,^42^ and Wang et al.,^43^ reported that foliar process and seed treatment method with 100-300 mg L^-1^ ZnO NPs knowingly enhanced okra growth and yield characteristics. Similar trends were also detected in such crops is wheat, maize, and tomato, where ZnO NPs heightened physiological functional ability and stress tolerance at optimum dosages. These optimistic effects are mostly attributed to enhanced Zn nutrition, initiation of antioxidant defense structures, and hormonal parameter. Nevertheless, excessive NPs acquaintance led to oxidative stress and growth inhibition, demonstrating that attentiveness and synthesis technique disapprovingly regulate ZnO NPs bio-activity and plant response.

### Metabolite identification and structure elucidation of metabolites

**Fig. 6:**
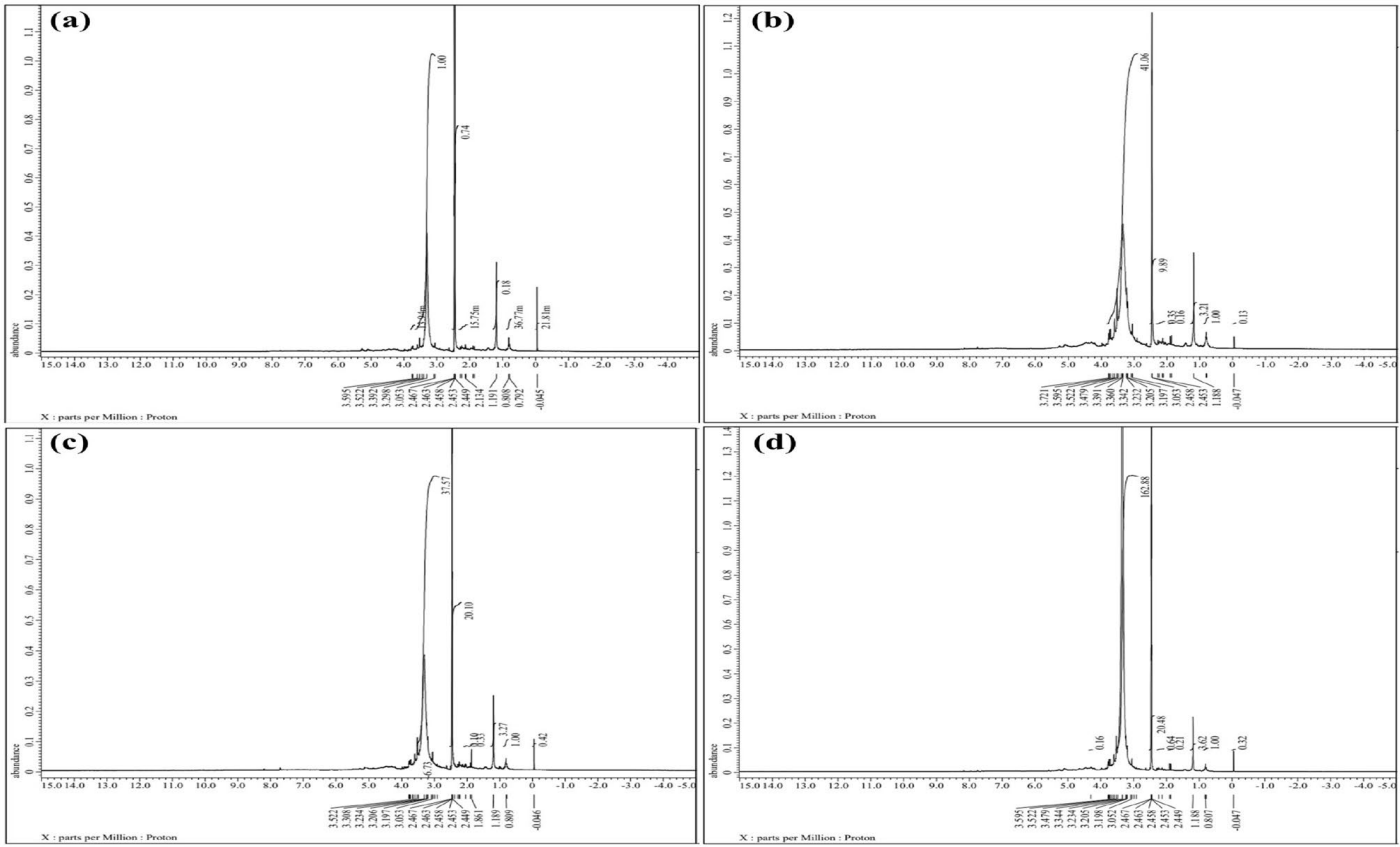
Graphical representation of ^1^H NMR analysis of *Abelmoschus esculantus* (L.) fruits (**a**) control sample; (**b**) 50ppm treatment; (**c**) 75ppm treatment; (d) 100ppm treatment.

The metabolomic analysis, metabolite identification is both the most crucial and time consuming phase. Assigning resonances and comparing the NMR analysis chemical shifts and coupling factors with those of real samples which are measured under the identical circumstances are the initial steps in the identification of metabolites^44^. Most plant materials contain roughly 20 common metabolites like α-Glucose, Amino acid Alanine, Glutamate, Glutamine & Threonine etc.^45^. Most of the time, ^1^H-NMR is adequate to produce metabolic statistics of a sample in a reasonable amount of time (5-10 min for 64-128 scans). A representative ^1^H-NMR spectrum data of plant material comprises more than a few hundreds of signals. Because the NMR signals are exactly proportionate to the molar concentration regardless of a compound’s physical characteristics, it reflects the total amount of metabolites in the extract^46^. The slope of the linear correlation via the indicated origin is equal to 0.991, with a 0.05 ppm rms error. The labile protons of the acids, amides, and diols have the worst mistakes. This is frequently caused by the protons strong concentration dependency, which results from hydrogen bonds between molecules^47^. ^1^H NMR spectra of *Abelmoschus esculentus* pod extract shown in Figure 6 (a), (b), (c) and (d). Majority of primary metabolites have distinct different signals, with amino acids that’s appearing between δ 2-0.5, at range of 3-2 δ organic, and sugars between δ 3-4^48^.

There are numerous distinctive indicators of secondary metabolites (SMs) in the aromatic area. Here are a few instances such as Indole alkaloids, including *Curcuzederone, catharanthine, vindoline, and phenylpropanoids; flavonoids; aliphatic glycosinolates*; and indole compounds, including *glucosinolate (neoglucobrassicin*) and indole acetic acid as well as γ*-aminobutyrate, β-turmerone, Selina-4(15),7(11)-dien-8-one* etc. in *Abelmoschus esculantus* (L.) fruits pods (Table 2). *Abelmoschus esculentus* showed a large transformation in their metabolite profiles that’s observed, not only their concentrations of individuals metabolites but also in the type’s derivatives, multiple signals, giving significantly clearer or sharper signals and thus noticeably facilitating peak identified^49^. As result the identification and quantification of SMs with NMR are as follows (Table 2). The Quercetin 3-O-glucosyl proton signal in the NMR spectra of *Abelmoschus esculentus* pod extract and also that present in all concentration of pod extract and Xanthorrhizol and its derivative proton signal in the ^1^H-NMR spectra between δ 0.808 to a range of 1.89 δ is shown in concentration of 50ppm-100pmm but its absent in controlled sample. The *Glutamine, α-Glucose, Threonine, Quercetin 3-O-glucosyl, Phosphocholine, α-D-Glucose*, show at different proton signal in ^1^H-^1^H J-resolved δH at 2.45ppm, 3.72ppm, 3.52ppm, 1.19ppm, 3.23ppm, and 3.69-3.39ppm respectively. The *Camphor, β-turmerone, 1-Bromopropane & Caprolactam* common metabolites that’s present in *Curcuma caesia* that’s also present in Pod fruits extract which shown by the proton signal of Me-1, ^1^H, ^1^H and 7H appeared at δH 0.809ppm, 2.134ppm, 3.522ppm and 3.052ppm as singlet because it did not have a significant neighboring proton atom (Table 3).

**Table 2:** ^1^H NMR chemical shifts of different solutes present in sample with reference δ-DMSO.

**Δ-DMSO**				
**Samples**	**Compound**	**Proton**	**Calculated**	**Observed Peaks (m)**
Control	-	-	-0.045	21.81
	*n-Pentane*	Me	0.792	36.77
	*n-Hexane*		0.808	
	*endo-2-Aminonorbornane*	7a	1.19	0.18
	*Acetaldehyde*	Me	2.134	15.75
	*2-Butanone*	CH_2_	2.449	0.74
	*n-Butylthiol*	1	2.458	
	*n-Propylamine*	1	2.463	
	*4-Methyl-N-acetylpiperidine*	6a	2.467	
	*Caprolactam*	7	3.053	1.00
	*Quebrachitol (4eq–2ax)*	3	3.298	
	*Diethylether*	CH_2_	3.392	13.94
	*THP*	2,6	3.522	
	*Diisopropylether*	CH	3.595	
Sample-1 (50ppm)	*-*	-	-0.047	0.13
	*cis,cis,cis-1,3,5-Cyclohexane triol (eq)*	2e/4e/6e	1.88	1.00
	*2-Butanone*	CH_2_	2.453	3.21
	*Acetophenone*	Me	2.458	
	*Caprolactam*	7	3.053	0.16
	*Methanol*	Me	3.197	
	*2-Pyrrolidinone*	5	3.205	0.35
	*N-Methyl valerolactam*	6	3.232	
	*Propanol*	1	3.342	9.89
	*trans-Cyclohexane-1,4-diol*	1,4	3.360	
	*1, 4-Butanediol*	1,4	3.391	41.06
	*-*	-	3.479	
	*exo-2-Norborneol*	2n	3.522	
	*Dissopropylether*	CH	3.596	
	*myo-Inositol (1ax-5eq)*	1e	3.721	
Sample-2 (75ppm)	*-*	-	-0.046	0.42
	*Camphor*	Me-1	0.809	1.00
	*Ethyl acetate*	CO. Me	1.189	3.27
	*endo-2-Norborneol*	6n	1.861	0.33
	*N-Me acetamide*	NMe	2.449	0.10
	*Amylamine*	1	2.453	20.10
	*n-Butylamine*	1	2.458	
	*Triethylamine*	Me	2.463	
	*Acetophenone*		2.467	
	*4-tBucyclohexylamine(cis)*	1e	3.053	6.73
	*2-Pyrrolidinone*	5	3.197	
	*N-Methyl valerolactam*	6	3.206	
			3.237	37.57
	*Propanol*	1	3.308	
	*1-Bromopropane*	1	3.522	
Sample-3 (100ppm)	*-*	-	-0.047	0.32
	*Camphor*	Me-1	0.807	1.00
	*Ethyl acetate*	CO. Me	1.188	3.62
	*N-Me acetamide*	NMe	2.449	
	*Amylamine*	1	2.453	0.21
	*n-Butylamine*	1	2.458	0.64
	*Acetophenone*	Me	2.463	
			2.467	
	*Caprolactam*	7	3.052	20.48
	*Methanol*	Me	3.198	
	*2-Pyrrolidinone*	5	3.205	
	*N-Methyl valerolactam*	6	3.234	162.88
	*N-Methyl caprolactam*	7	3.344	
	*Bromoethane*	CH_2_	3.479	0.16
	*exo-2-Norborneol*	2n	3.522	
	*(C)-chiro-Inositol (4eq–2ax)*	1.6	3.595

**Table 3:** Identification of ^1^H NMR metabolites in dried extract of *Abelmoschus esculantus* (L.) fruits after ZnO NPs treatment and as well as control sample.

**Metabolite**	^**1**^**H NMR signal δ/ppm**	**Sample concentration (ppm)**			
		**Control**	**50ppm**	**75ppm**	**100ppm**
-	-0.04	-	-	-	-
*Curdione*	0.808	-	+	+	+
*Amadannulen*		-	+	+	+
*Quercetin 3-O-glucosyl*	1.19	+	+	+	+
*Selina-4(15),7(11)-dien-8-one*		-	-	+	+
*Zedoalactone*		-	-	+	+
*Zedoardiol*		-	-	+	+
*Curcuzederone*	1.182	-	-	+	+
*Xanthorrhizol derivative*	1.188	-	-	+	+
*β-turmerone*	1.86	-	+	+	+
*Xanthorrhizol*		-	+	+	+
*β-turmerone*	2.134	+	-	+	+
*Zerumin-B*	2.449	+	-	+	+
*Succinate*	2.453	-	+	+	+
*Glutamine*	2.458	+	+	+	+
*Zerumin-B*	2.463	-	+	+	+
*γ-aminobutyrate*	3.050	+	+	+	+
	3.053	+	+	+	+
*Choline*	3.19	+	+	+	+
*Choline*	3.20	+	+	+	+
*Phosphocholine*	3.23	+	+	+	-
*Alanine*	3.29	+	-	-	+
*-*	3.30	-	-	-	-
*α-Glucose*	3.34	+	+	+	+
*α-D-Glucose*	3.36	+	+	+	+
*α-D-Glucose*	3.39	+	-	+	+
*Sucrose*	3.47	+	+	-	-
*Threonine*	3.52	+	+	+	+
*Sucrose*	3.59	+	+	+	+
*α-Glucose*	3.72	+	+	+	+
*Curcuzederone*		-	+	-	+

ZnO NPs impact SMs mostly through two mechanisms that’s: (i) gradual proclamation of Zn^2+^ ions, which assist as indispensable micronutrients and enzyme cofactors, and (ii) elicitation of insignificant oxidative and signaling rejoinders at the cellular level. Zn is a significant cofactor for enzymes complicated in the shikimate and phenylpropanoid alleyways, which regulate phenolic, flavonoid, and lignin biosynthesis^50^. Consequently, heightened gathering of phenolic acids, flavonoids, and associated metabolites underneath ZnO NPs treatment imitates stimulation of antioxidant and defense pathways. Furthermore, ZnO NPs can able to encourage reactive oxygen species (ROS) peer group at low absorptions, energizing SMs biosynthesis via jasmonic acid, salicylic acid, as well as MAPK signaling cascades^51^. Upregulated amino acid byproducts such as tryptophan and tyrosine metabolites propose augmented flux in the direction of indole alkaloids and phenylpropanoids, although fluctuations in terpenoid side view designate inflection of the isoprenoid pathway, contributive to stress tolerance, sheath stabilization, and defense^52^.

## Conclusion

In this study, ZnO-NPs were green synthesized from rhizome extract of *Curcuma caesia* and employed to enhance growth a metabolic response in *Abelmoschus esculentus*. The biosynthesized ZnO NPs were found to be crystalline in nature, relatively stable dispersion and characteristic Zn-O bonding by various physicochemical characterization studies. The biological evaluation indicated concentration-dependent response with low to moderate ZnO NP doses resulting into stimulatory effect on germination, vegetative growth, flowering and yield parameters and higher concentrations leading to a differential physiological response. Metabolomic profiling based on ^1H-NMR also revealed the ZnO NP–induced modulation of primary and secondary metabolites related to plant growth, nutrient use and stress. In general, it can be inferred that plant-mediated ZnO NPs may be a potent nanomaterial for better crop production if used at an appropriate dose. The present study offers a mechanistic understanding of the relationship between green-synthesized ZnO NPs and plant physiological processes, to serve as an ecological perspective into their usage within sustainable farming contexts.

## Supplementary Information

Supplementary Information.

## Data Availability

Data will be made available on request from the corresponding authors.
