# Supplementary material for: Synthesis and characterization of Curcuma Caesia plant root extract-mediated ZnO nanoparticles: efficacy as soil conditioner and plant growth promoter
Source: Sci Rep. 2026 Apr 22;16:13050. doi: 10.1038/s41598-026-41196-w (PMC13100065; doi:10.1038/s41598-026-41196-w)
Supplement: Supplementary file 1 — Supplementary Information. [file 41598_2026_41196_MOESM1_ESM.docx]

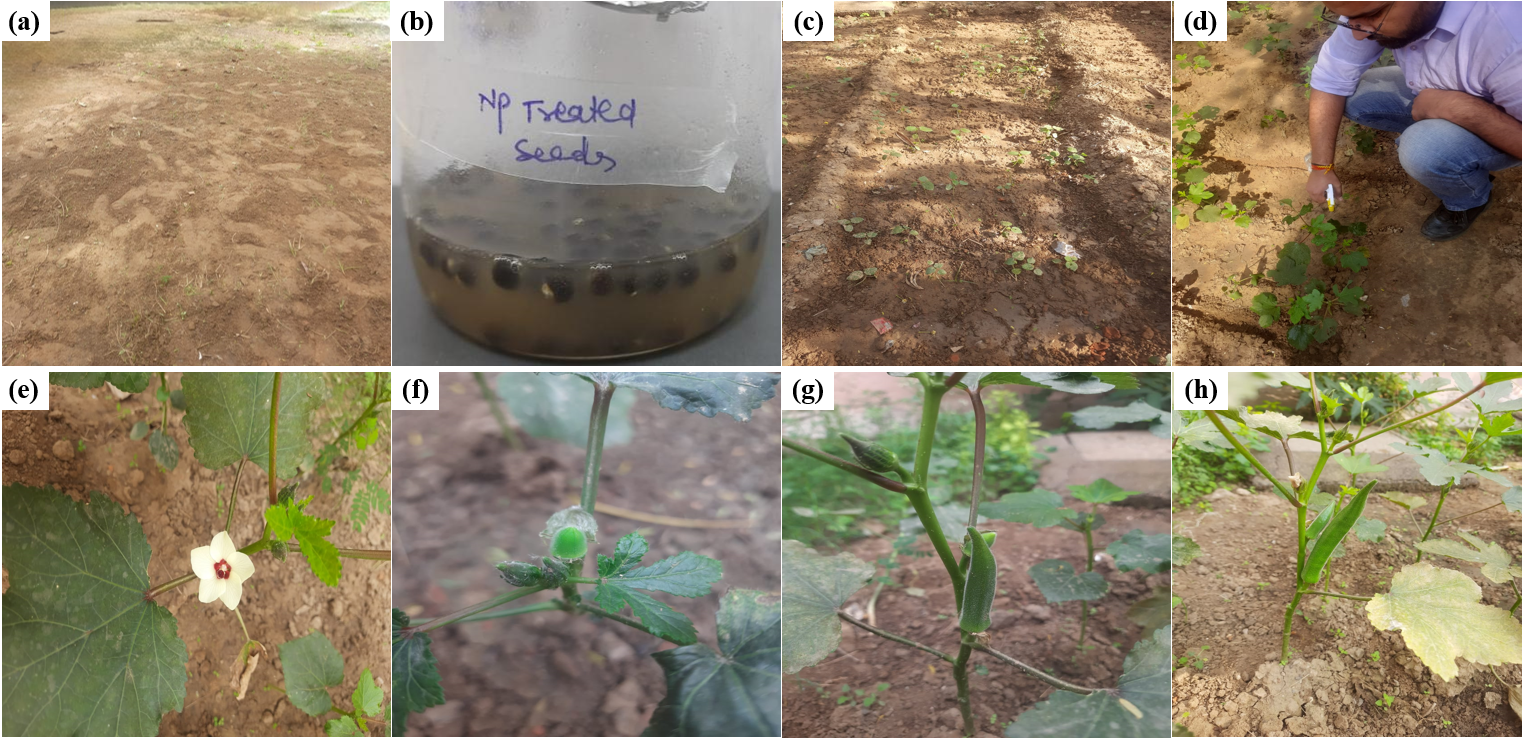


**Supplementary Figure 1:** **(a) Shown agriculture field for planting, (b) nanoparticle treated okra seeds, (c) okra seed germinated (two leaves), (d) foliar spraying of NPs on plants, (e) flowering on plant, (f) fruit budding on plant, (g) pre-mature fruit of okra, (h) matured fruit of okra after treatment.**

**Supplementary Table 1: Mean values of experimental observed data of the excess effect of ZnO NPs on the growth and yield of *Abelmoschus esculentus* crop variety Bhindi Pusa 5 (DOV 66).**

| **Treatment** | **Germination Percentage** | **Plants height (cm)** | **Leaf length**  **(cm)** | **Leaf width (cm)** | **Flowering %** | **Number of pods per crop** | **Pod weight (grams)** | **Seed’s quantity**  **(Avg.)** |
| --- | --- | --- | --- | --- | --- | --- | --- | --- |
| **Control** | 71.0000 | 13.2667 | 6.8333 | 8.7000 | 51.6000 | 4.0000 | 10.4300 | 30 |
| **T1 (50ppm)** | 94.6333 | 19.4667 | 9.6333 | 12.2000 | 71.0767 | 8.0000 | 23.1100 | 82 |
| **T2 (75ppm)** | 92.8533 | 15.3667 | 9.4000 | 10.8000 | 38.4000 | 6.0000 | 26.9667 | 97 |
| **T3 (100ppm)** | 85.5333 | 23.4467 | 12.4000 | 12.9700 | 88.4000 | 16.0000 | 24.1333 | 91 |

**ANOVA**

**Supplementary Table 2: Descriptive Two-Way Anova analysis of germination percentage of *Abelmoschus esculantus*** **crop variety Bhindi Pusa 5 (DOV 66) at 0.05 significant level on least standard deviation.**

| **Descriptives** | | | | | | | | |
| --- | --- | --- | --- | --- | --- | --- | --- | --- |
|  | **N** | **Mean** | **Std. Deviation** | **Std. Error** | **95% Confidence Interval for Mean** | | **Minimum** | **Maximum** |
|  |  |  |  |  | **Lower Bound** | **Upper Bound** |  |  |
| Control | 3 | 71.0000 | .95394 | .55076 | 68.6303 | 73.3697 | 70.10 | 72.00 |
| 50ppm | 3 | 94.6333 | .55076 | .31798 | 93.2652 | 96.0015 | 94.00 | 95.00 |
| 75ppm | 3 | 92.8533 | .50163 | .28962 | 91.6072 | 94.0995 | 92.33 | 93.33 |
| 100ppm | 3 | 85.5333 | .92376 | .53333 | 83.2386 | 87.8281 | 85.00 | 86.60 |
| Total | 12 | 86.0050 | 9.74585 | 2.81338 | 79.8128 | 92.1972 | 70.10 | 95.00 |

| **Supplementary Table 2.1: Multiple comparison with dependent variables of germination percentage of *Abelmoschus esculantus* crop variety Bhindi Pusa 5 (DOV 66) at 0.05 significant level.** | | | | | | | |
| --- | --- | --- | --- | --- | --- | --- | --- |
| **Multiple Comparisons** | | | | | | | |
| **Dependent Variable: Germination Percentage** | | | | | | | |
|  | **(I) Treatment** | **(J) Treatment** | **Mean Difference (I-J)** | **Std. Error** | **Sig.** | **95% Confidence Interval** | |
|  |  |  |  |  |  | **Lower Bound** | **Upper Bound** |
| LSD | Control | 50ppm | -23.63333^*^ | .62160 | .000 | -25.0667 | -22.1999 |
|  |  | 75ppm | -21.85333^*^ | .62160 | .000 | -23.2867 | -20.4199 |
|  |  | 100ppm | -14.53333^*^ | .62160 | .000 | -15.9667 | -13.0999 |
|  | 50ppm | Control | 23.63333^*^ | .62160 | .000 | 22.1999 | 25.0667 |
|  |  | 75ppm | 1.78000^*^ | .62160 | .021 | .3466 | 3.2134 |
|  |  | 100ppm | 9.10000^*^ | .62160 | .000 | 7.6666 | 10.5334 |
|  | 75ppm | Control | 21.85333^*^ | .62160 | .000 | 20.4199 | 23.2867 |
|  |  | 50PPM | -1.78000^*^ | .62160 | .021 | -3.2134 | -.3466 |
|  |  | 100PPM | 7.32000^*^ | .62160 | .000 | 5.8866 | 8.7534 |
|  | 100ppm | Control | 14.53333^*^ | .62160 | .000 | 13.0999 | 15.9667 |
|  |  | 50ppm | -9.10000^*^ | .62160 | .000 | -10.5334 | -7.6666 |
|  |  | 75ppm | -7.32000^*^ | .62160 | .000 | -8.7534 | -5.8866 |
| Dunnett T3 | Control | 50ppm | -23.63333^*^ | .63596 | .000 | -26.8124 | -20.4543 |
|  |  | 75ppm | -21.85333^*^ | .62226 | .000 | -25.0882 | -18.6185 |
|  |  | 100ppm | -14.53333^*^ | .76667 | .000 | -17.8825 | -11.1841 |
|  | 50ppm | control | 23.63333^*^ | .63596 | .000 | 20.4543 | 26.8124 |
|  |  | 75ppm | 1.78000 | .43010 | .060 | -.1066 | 3.6666 |
|  |  | 100ppm | 9.10000^*^ | .62093 | .002 | 6.0364 | 12.1636 |
|  | 75ppm | control | 21.85333^*^ | .62226 | .000 | 18.6185 | 25.0882 |
|  |  | 50ppm | -1.78000 | .43010 | .060 | -3.6666 | .1066 |
|  |  | 100ppm | 7.32000^*^ | .60690 | .004 | 4.2077 | 10.4323 |
|  | 100ppm | control | 14.53333^*^ | .76667 | .000 | 11.1841 | 17.8825 |
|  |  | 50ppm | -9.10000^*^ | .62093 | .002 | -12.1636 | -6.0364 |
|  |  | 75ppm | -7.32000^*^ | .60690 | .004 | -10.4323 | -4.2077 |
| ***. The mean difference is significant at the 0.05 level.** | | | | | | | |

**Supplementary Table 2.2: Means for groups in homogeneous subsets are displayed of germination percentage of *Abelmoschus esculantus.***

| **Germination Percentage** | | | | | | |
| --- | --- | --- | --- | --- | --- | --- |
| **Duncan^a^** | **Treatment** | **N** | **Subset for alpha = 0.05** | | | |
|  |  |  | **1** | **2** | **3** | **4** |
|  | Control | 3 | 71.0000 |  |  |  |
|  | 100PPM | 3 |  | 85.5333 |  |  |
|  | 75PPM | 3 |  |  | 92.8533 |  |
|  | 50PPM | 3 |  |  |  | 94.6333 |
|  | Sig. |  | 1.000 | 1.000 | 1.000 | 1.000 |
| **Means for groups in homogeneous subsets are displayed.** | | | | | | |
| **a. Uses Harmonic Mean Sample Size = 3.000.** | | | | | | |

**Supplementary Table 3: Measurement and descriptive mean value analysis of plant height of *Abelmoschus esculantus* crop variety Bhindi Pusa 5 (DOV 66) at 0.05 significant level.**

| **Descriptives** | | | | | | | | |
| --- | --- | --- | --- | --- | --- | --- | --- | --- |
|  | **N** | **Mean** | **Std. Deviation** | **Std. Error** | **95% Confidence Interval for Mean** | | **Minimum** | **Maximum** |
|  |  |  |  |  | **Lower Bound** | **Upper Bound** |  |  |
| Control | 3 | 13.2667 | .55076 | .31798 | 11.8985 | 14.6348 | 12.90 | 13.90 |
| 50ppm | 3 | 19.4667 | .50332 | .29059 | 18.2163 | 20.7170 | 19.00 | 20.00 |
| 75ppm | 3 | 15.3667 | .56862 | .32830 | 13.9541 | 16.7792 | 14.90 | 16.00 |
| 100ppm | 3 | 23.4467 | .50846 | .29356 | 22.1836 | 24.7098 | 23.00 | 24.00 |
| Total | 12 | 17.8867 | 4.10755 | 1.18575 | 15.2769 | 20.4965 | 12.90 | 24.00 |

| **Supplementary Table 3.1: Multiple comparison of dependent variables of plant height of *Abelmoschus esculantus* crop variety Bhindi Pusa 5 (DOV 66) at 0.05 significant level.** | | | | | | | |
| --- | --- | --- | --- | --- | --- | --- | --- |
| **Multiple Comparisons** | | | | | | | |
| **Dependent Variable: Plant height** | | | | | | | |
|  | **(I) Treatment** | **(J) Treatment** | **Mean Difference (I-J)** | **Std. Error** | **Sig.** | **95% Confidence Interval** | |
|  |  |  |  |  |  | **Lower Bound** | **Upper Bound** |
| LSD | Control | 50ppm | -6.20000^*^ | .43561 | .000 | -7.2045 | -5.1955 |
|  |  | 75ppm | -2.10000^*^ | .43561 | .001 | -3.1045 | -1.0955 |
|  |  | 100ppm | -10.18000^*^ | .43561 | .000 | -11.1845 | -9.1755 |
|  | 50ppm | Control | 6.20000^*^ | .43561 | .000 | 5.1955 | 7.2045 |
|  |  | 75ppm | 4.10000^*^ | .43561 | .000 | 3.0955 | 5.1045 |
|  |  | 100ppm | -3.98000^*^ | .43561 | .000 | -4.9845 | -2.9755 |
|  | 75ppm | Control | 2.10000^*^ | .43561 | .001 | 1.0955 | 3.1045 |
|  |  | 50ppm | -4.10000^*^ | .43561 | .000 | -5.1045 | -3.0955 |
|  |  | 100ppm | -8.08000^*^ | .43561 | .000 | -9.0845 | -7.0755 |
|  | 100ppm | Control | 10.18000^*^ | .43561 | .000 | 9.1755 | 11.1845 |
|  |  | 50ppm | 3.98000^*^ | .43561 | .000 | 2.9755 | 4.9845 |
|  |  | 75ppm | 8.08000^*^ | .43561 | .000 | 7.0755 | 9.0845 |
| Dunnett T3 | Control | 50ppm | -6.20000^*^ | .43076 | .001 | -8.0889 | -4.3111 |
|  |  | 75ppm | -2.10000^*^ | .45704 | .042 | -4.0966 | -.1034 |
|  |  | 100ppm | -10.18000^*^ | .43277 | .000 | -12.0759 | -8.2841 |
|  | 50ppm | Control | 6.20000^*^ | .43076 | .001 | 4.3111 | 8.0889 |
|  |  | 75ppm | 4.10000^*^ | .43843 | .003 | 2.1706 | 6.0294 |
|  |  | 100ppm | -3.98000^*^ | .41306 | .003 | -5.7836 | -2.1764 |
|  | 75ppm | Control | 2.10000^*^ | .45704 | .042 | .1034 | 4.0966 |
|  |  | 50ppm | -4.10000^*^ | .43843 | .003 | -6.0294 | -2.1706 |
|  |  | 100ppm | -8.08000^*^ | .44040 | .000 | -10.0156 | -6.1444 |
|  | 100ppm | Control | 10.18000^*^ | .43277 | .000 | 8.2841 | 12.0759 |
|  |  | 50ppm | 3.98000^*^ | .41306 | .003 | 2.1764 | 5.7836 |
|  |  | 75ppm | 8.08000^*^ | .44040 | .000 | 6.1444 | 10.0156 |
| ***. The mean difference is significant at the 0.05 level.** | | | | | | | |

**Supplementary Table 3.2: Means for groups in homogeneous subsets are displayed of plant height of *Abelmoschus esculantus.***

| **Plant height** | | | | | | |
| --- | --- | --- | --- | --- | --- | --- |
| **Duncan^a^** | **Treatment** | **N** | **Subset for alpha = 0.05** | | | |
|  |  |  | **1** | **2** | **3** | **4** |
|  | Control | 3 | 13.2667 |  |  |  |
|  | 75ppm | 3 |  | 15.3667 |  |  |
|  | 50ppm | 3 |  |  | 19.4667 |  |
|  | 100ppm | 3 |  |  |  | 23.4467 |
|  | Sig. |  | 1.000 | 1.000 | 1.000 | 1.000 |
| **Means for groups in homogeneous subsets are displayed.** | | | | | | |
| **a. Uses Harmonic Mean Sample Size = 3.000.** | | | | | | |

**Supplementary Table 4: Descriptive measurement and mean value of individual plant leaves length of *Abelmoschus esculantus* crop variety Bhindi Pusa 5 (DOV 66) at 0.05 significant level.**

| **Descriptives** | | | | | | | | |
| --- | --- | --- | --- | --- | --- | --- | --- | --- |
|  | **N** | **Mean** | **Std. Deviation** | **Std. Error** | **95% Confidence Interval for Mean** | | **Minimum** | **Maximum** |
|  |  |  |  |  | **Lower Bound** | **Upper Bound** |  |  |
| Control | 3 | 6.8333 | .30551 | .17638 | 6.0744 | 7.5922 | 6.50 | 7.10 |
| 50ppm | 3 | 9.6333 | .37859 | .21858 | 8.6929 | 10.5738 | 9.20 | 9.90 |
| 75ppm | 3 | 9.4000 | .10000 | .05774 | 9.1516 | 9.6484 | 9.30 | 9.50 |
| 100ppm | 3 | 12.4000 | .45826 | .26458 | 11.2616 | 13.5384 | 12.00 | 12.90 |
| Total | 12 | 9.5667 | 2.07817 | .59992 | 8.2463 | 10.8871 | 6.50 | 12.90 |

| **Supplementary Table 4.1: Multiple comparisons with dependent variables of individual plant leaves length of *Abelmoschus esculantus* crop variety Bhindi Pusa 5 (DOV 66) at 0.05 significant level.** | | | | | | | |
| --- | --- | --- | --- | --- | --- | --- | --- |
| **Multiple Comparisons** | | | | | | | |
| **Dependent Variable: Leaf length** | | | | | | | |
|  | **(I) Treatment** | **(J) Treatment** | **Mean Difference (I-J)** | **Std. Error** | **Sig.** | **95% Confidence Interval** | |
|  |  |  |  |  |  | **Lower Bound** | **Upper Bound** |
| LSD | Control | 50ppm | -2.80000^*^ | .27588 | .000 | -3.4362 | -2.1638 |
|  |  | 75ppm | -2.56667^*^ | .27588 | .000 | -3.2029 | -1.9305 |
|  |  | 100ppm | -5.56667^*^ | .27588 | .000 | -6.2029 | -4.9305 |
|  | 50ppm | Control | 2.80000^*^ | .27588 | .000 | 2.1638 | 3.4362 |
|  |  | 75ppm | .23333 | .27588 | .422 | -.4029 | .8695 |
|  |  | 100ppm | -2.76667^*^ | .27588 | .000 | -3.4029 | -2.1305 |
|  | 75ppm | Control | 2.56667^*^ | .27588 | .000 | 1.9305 | 3.2029 |
|  |  | 50ppm | -.23333 | .27588 | .422 | -.8695 | .4029 |
|  |  | 100ppm | -3.00000^*^ | .27588 | .000 | -3.6362 | -2.3638 |
|  | 100ppm | Control | 5.56667^*^ | .27588 | .000 | 4.9305 | 6.2029 |
|  |  | 50ppm | 2.76667^*^ | .27588 | .000 | 2.1305 | 3.4029 |
|  |  | 75ppm | 3.00000^*^ | .27588 | .000 | 2.3638 | 3.6362 |
| Dunnett T3 | Control | 50ppm | -2.80000^*^ | .28087 | .003 | -4.0560 | -1.5440 |
|  |  | 75ppm | -2.56667^*^ | .18559 | .008 | -3.7267 | -1.4066 |
|  |  | 100ppm | -5.56667^*^ | .31798 | .001 | -7.0702 | -4.0632 |
|  | 50ppm | Control | 2.80000^*^ | .28087 | .003 | 1.5440 | 4.0560 |
|  |  | 75ppm | .23333 | .22608 | .851 | -1.2677 | 1.7343 |
|  |  | 100ppm | -2.76667^*^ | .34319 | .006 | -4.2939 | -1.2394 |
|  | 75ppm | Control | 2.56667^*^ | .18559 | .008 | 1.4066 | 3.7267 |
|  |  | 50ppm | -.23333 | .22608 | .851 | -1.7343 | 1.2677 |
|  |  | 100ppm | -3.00000^*^ | .27080 | .018 | -4.8721 | -1.1279 |
|  | 100ppm | Control | 5.56667^*^ | .31798 | .001 | 4.0632 | 7.0702 |
|  |  | 50ppm | 2.76667^*^ | .34319 | .006 | 1.2394 | 4.2939 |
|  |  | 75ppm | 3.00000^*^ | .27080 | .018 | 1.1279 | 4.8721 |
| ***. The mean difference is significant at the 0.05 level.** | | | | | | | |

**Supplementary Table 4.2: Means for groups in homogeneous subsets are displayed of Leaf length of *Abelmoschus esculantus.***

| **Leaf length** | | | | | |
| --- | --- | --- | --- | --- | --- |
| **Duncan^a^** | **Treatment** | **N** | **Subset for alpha = 0.05** | | |
|  |  |  | **1** | **2** | **3** |
|  | Control | 3 | 6.8333 |  |  |
|  | 75ppm | 3 |  | 9.4000 |  |
|  | 50ppm | 3 |  | 9.6333 |  |
|  | 100ppm | 3 |  |  | 12.4000 |
|  | Sig. |  | 1.000 | .422 | 1.000 |
| **Means for groups in homogeneous subsets are displayed.** | | | | | |
| **a. Uses Harmonic Mean Sample Size = 3.000.** | | | | | |

**Supplementary Table 5: Descriptive measurement and mean value analysis of plant leaves width of *Abelmoschus esculantus* crop variety Bhindi Pusa 5 (DOV 66) at 0.05 significant level and 95% Confidence Interval for Mean.**

| **Descriptives** | | | | | | | | |
| --- | --- | --- | --- | --- | --- | --- | --- | --- |
|  | **N** | **Mean** | **Std. Deviation** | **Std. Error** | **95% Confidence Interval for Mean** | | **Minimum** | **Maximum** |
|  |  |  |  |  | **Lower Bound** | **Upper Bound** |  |  |
| Control | 3 | 8.7000 | .20000 | .11547 | 8.2032 | 9.1968 | 8.50 | 8.90 |
| 50ppm | 3 | 12.2000 | .36056 | .20817 | 11.3043 | 13.0957 | 11.90 | 12.60 |
| 75ppm | 3 | 10.8000 | .26458 | .15275 | 10.1428 | 11.4572 | 10.50 | 11.00 |
| 100ppm | 3 | 12.9700 | .06083 | .03512 | 12.8189 | 13.1211 | 12.90 | 13.01 |
| Total | 12 | 11.1675 | 1.70836 | .49316 | 10.0821 | 12.2529 | 8.50 | 13.01 |

| **Supplementary Table 5.1: Multiple comparison with dependent variables of plant leaves width of *Abelmoschus esculantus* crop variety Bhindi Pusa 5 (DOV 66) at 95% Confidence Interval for Mean.** | | | | | | | |
| --- | --- | --- | --- | --- | --- | --- | --- |
| **Multiple Comparisons** | | | | | | | |
| **Dependent Variable: Leaves width** | | | | | | | |
|  | **(I) Treatment** | **(J) Treatment** | **Mean Difference (I-J)** | **Std. Error** | **Sig.** | **95% Confidence Interval** | |
|  |  |  |  |  |  | **Lower Bound** | **Upper Bound** |
| LSD | Control | 50ppm | -3.50000^*^ | .20154 | .000 | -3.9647 | -3.0353 |
|  |  | 75ppm | -2.10000^*^ | .20154 | .000 | -2.5647 | -1.6353 |
|  |  | 100ppm | -4.27000^*^ | .20154 | .000 | -4.7347 | -3.8053 |
|  | 50ppm | Control | 3.50000^*^ | .20154 | .000 | 3.0353 | 3.9647 |
|  |  | 75ppm | 1.40000^*^ | .20154 | .000 | .9353 | 1.8647 |
|  |  | 100ppm | -.77000^*^ | .20154 | .005 | -1.2347 | -.3053 |
|  | 75ppm | Control | 2.10000^*^ | .20154 | .000 | 1.6353 | 2.5647 |
|  |  | 50ppm | -1.40000^*^ | .20154 | .000 | -1.8647 | -.9353 |
|  |  | 100ppm | -2.17000^*^ | .20154 | .000 | -2.6347 | -1.7053 |
|  | 100ppm | Control | 4.27000^*^ | .20154 | .000 | 3.8053 | 4.7347 |
|  |  | 50ppm | .77000^*^ | .20154 | .005 | .3053 | 1.2347 |
|  |  | 75ppm | 2.17000^*^ | .20154 | .000 | 1.7053 | 2.6347 |
| Dunnett T3 | Control | 50ppm | -3.50000^*^ | .23805 | .002 | -4.7099 | -2.2901 |
|  |  | 75ppm | -2.10000^*^ | .19149 | .002 | -2.9701 | -1.2299 |
|  |  | 100ppm | -4.27000^*^ | .12069 | .001 | -5.0416 | -3.4984 |
|  | 50ppm | Control | 3.50000^*^ | .23805 | .002 | 2.2901 | 4.7099 |
|  |  | 75ppm | 1.40000^*^ | .25820 | .029 | .2168 | 2.5832 |
|  |  | 100ppm | -.77000 | .21111 | .185 | -2.2861 | .7461 |
|  | 75ppm | Control | 2.10000^*^ | .19149 | .002 | 1.2299 | 2.9701 |
|  |  | 50ppm | -1.40000^*^ | .25820 | .029 | -2.5832 | -.2168 |
|  |  | 100ppm | -2.17000^*^ | .15674 | .011 | -3.2429 | -1.0971 |
|  | 100ppm | Control | 4.27000^*^ | .12069 | .001 | 3.4984 | 5.0416 |
|  |  | 50ppm | .77000 | .21111 | .185 | -.7461 | 2.2861 |
|  |  | 75ppm | 2.17000^*^ | .15674 | .011 | 1.0971 | 3.2429 |
| ***. The mean difference is significant at the 0.05 level.** | | | | | | | |

**Supplementary Table 5.2: Means for groups in homogeneous subsets are displayed of plant leaves width of *Abelmoschus esculantus.***

| **Leaves width** | | | | | | |
| --- | --- | --- | --- | --- | --- | --- |
| **Duncan^a^** | **Treatment** | **N** | **Subset for alpha = 0.05** | | | |
|  |  |  | **1** | **2** | **3** | **4** |
|  | Control | 3 | 8.7000 |  |  |  |
|  | 75ppm | 3 |  | 10.8000 |  |  |
|  | 50ppm | 3 |  |  | 12.2000 |  |
|  | 100ppm | 3 |  |  |  | 12.9700 |
|  | Sig. |  | 1.000 | 1.000 | 1.000 | 1.000 |
| **Means for groups in homogeneous subsets are displayed.** | | | | | | |
| **a. Uses Harmonic Mean Sample Size = 3.000.** | | | | | | |

**Supplementary Table 6: Descriptive mean value analysis of flowering percentage of *Abelmoschus esculantus* crop variety Bhindi Pusa 5 (DOV 66) at 0.05 significant level.**

| **Descriptives** | | | | | | | | | |
| --- | --- | --- | --- | --- | --- | --- | --- | --- | --- |
|  | **N** | **Mean** | **Std. Deviation** | **Std. Error** | **95% Confidence Interval for Mean** | | **Minimum** | **Maximum** |  |
|  |  |  |  |  | **Lower Bound** | **Upper Bound** |  |  |  |
| Control | 3 | 51.6000 | .70000 | .40415 | 49.8611 | 53.3389 | 50.90 | 52.30 |  |
| 50ppm | 3 | 71.0767 | .97500 | .56292 | 68.6546 | 73.4987 | 70.00 | 71.90 |  |
| 75ppm | 3 | 38.4000 | .55678 | .32146 | 37.0169 | 39.7831 | 37.90 | 39.00 |  |
| 100ppm | 3 | 88.4000 | .55678 | .32146 | 87.0169 | 89.7831 | 87.90 | 89.00 |  |
| Total | 12 | 62.3692 | 19.85375 | 5.73128 | 49.7547 | 74.9836 | 37.90 | 89.00 |  |

| **Supplementary Table 6.1: Multiple comparison with dependent variables of *Abelmoschus esculantus* crop variety Bhindi Pusa 5 (DOV 66) flowering percentage at 0.05 significant level.** | | | | | | | |
| --- | --- | --- | --- | --- | --- | --- | --- |
| **Multiple Comparisons** | | | | | | | |
| **Dependent Variable: Flowering percentage** | | | | | | | |
|  | **(I) Treatment** | **(J) Treatment** | **Mean Difference (I-J)** | **Std. Error** | **Sig.** | **95% Confidence Interval** | |
|  |  |  |  |  |  | **Lower Bound** | **Upper Bound** |
| LSD | Control | 50ppm | -19.47667^*^ | .58604 | .000 | -20.8281 | -18.1253 |
|  |  | 75ppm | 13.20000^*^ | .58604 | .000 | 11.8486 | 14.5514 |
|  |  | 100ppm | -36.80000^*^ | .58604 | .000 | -38.1514 | -35.4486 |
|  | 50ppm | Control | 19.47667^*^ | .58604 | .000 | 18.1253 | 20.8281 |
|  |  | 75ppm | 32.67667^*^ | .58604 | .000 | 31.3253 | 34.0281 |
|  |  | 100ppm | -17.32333^*^ | .58604 | .000 | -18.6747 | -15.9719 |
|  | 75ppm | Control | -13.20000^*^ | .58604 | .000 | -14.5514 | -11.8486 |
|  |  | 50ppm | -32.67667^*^ | .58604 | .000 | -34.0281 | -31.3253 |
|  |  | 100ppm | -50.00000^*^ | .58604 | .000 | -51.3514 | -48.6486 |
|  | 100ppm | Control | 36.80000^*^ | .58604 | .000 | 35.4486 | 38.1514 |
|  |  | 50ppm | 17.32333^*^ | .58604 | .000 | 15.9719 | 18.6747 |
|  |  | 75ppm | 50.00000^*^ | .58604 | .000 | 48.6486 | 51.3514 |
| Dunnett T3 | Control | 50ppm | -19.47667^*^ | .69297 | .000 | -22.6731 | -16.2802 |
|  |  | 75ppm | 13.20000^*^ | .51640 | .000 | 10.8833 | 15.5167 |
|  |  | 100ppm | -36.80000^*^ | .51640 | .000 | -39.1167 | -34.4833 |
|  | 50ppm | Control | 19.47667^*^ | .69297 | .000 | 16.2802 | 22.6731 |
|  |  | 75ppm | 32.67667^*^ | .64824 | .000 | 29.4215 | 35.9318 |
|  |  | 100ppm | -17.32333^*^ | .64824 | .000 | -20.5785 | -14.0682 |
|  | 75ppm | Control | -13.20000^*^ | .51640 | .000 | -15.5167 | -10.8833 |
|  |  | 50ppm | -32.67667^*^ | .64824 | .000 | -35.9318 | -29.4215 |
|  |  | 100ppm | -50.00000^*^ | .45461 | .000 | -51.9849 | -48.0151 |
|  | 100ppm | Control | 36.80000^*^ | .51640 | .000 | 34.4833 | 39.1167 |
|  |  | 50ppm | 17.32333^*^ | .64824 | .000 | 14.0682 | 20.5785 |
|  |  | 75ppm | 50.00000^*^ | .45461 | .000 | 48.0151 | 51.9849 |
| ***. The mean difference is significant at the 0.05 level.** | | | | | | | |

**Supplementary Table 6.2: Means for groups in homogeneous subsets are displayed of flowering percentage width of plant *Abelmoschus esculantus.***

| **Flowering percentage** | | | | | | |
| --- | --- | --- | --- | --- | --- | --- |
| **Duncan^a^** | **Treatment** | **N** | **Subset for alpha = 0.05** | | | |
|  |  |  | **1** | **2** | **3** | **4** |
|  | 75ppm | 3 | 38.4000 |  |  |  |
|  | Control | 3 |  | 51.6000 |  |  |
|  | 50ppm | 3 |  |  | 71.0767 |  |
|  | 100ppm | 3 |  |  |  | 88.4000 |
|  | Sig. |  | 1.000 | 1.000 | 1.000 | 1.000 |
| **Means for groups in homogeneous subsets are displayed.** | | | | | | |
| **a. Uses Harmonic Mean Sample Size = 3.000.** | | | | | | |

**Supplementary Table 7: Descriptive mean value analysis of numbers of pods per crops of *Abelmoschus esculantus* crop variety Bhindi Pusa 5 (DOV 66) at 0.05 significant level.**

| **Descriptives** | | | | | | | | |
| --- | --- | --- | --- | --- | --- | --- | --- | --- |
|  | **N** | **Mean** | **Std. Deviation** | **Std. Error** | **95% Confidence Interval for Mean** | | **Minimum** | **Maximum** |
|  |  |  |  |  | **Lower Bound** | **Upper Bound** |  |  |
| Control | 3 | 4.0000 | 1.00000 | .57735 | 1.5159 | 6.4841 | 3.00 | 5.00 |
| 50ppm | 3 | 8.0000 | 1.00000 | .57735 | 5.5159 | 10.4841 | 7.00 | 9.00 |
| 75ppm | 3 | 6.0000 | 1.00000 | .57735 | 3.5159 | 8.4841 | 5.00 | 7.00 |
| 100ppm | 3 | 16.3333 | 7.09460 | 4.09607 | -1.2906 | 33.9573 | 10.00 | 24.00 |
| Total | 12 | 8.5833 | 5.80687 | 1.67630 | 4.8938 | 12.2728 | 3.00 | 24.00 |

| **Supplementary Table 7.1: Multiple comparisons with dependent variables of numbers of pods per crops of *Abelmoschus esculantus* crop variety Bhindi Pusa 5 (DOV 66) at 0.05 significant level.** | | | | | | | |
| --- | --- | --- | --- | --- | --- | --- | --- |
| **Multiple Comparisons** | | | | | | | |
| **Dependent Variable: Number of pods per crop** | | | | | | | |
|  | **(I) Treatment** | **(J) Treatment** | **Mean Difference (I-J)** | **Std. Error** | **Sig.** | **95% Confidence Interval** | |
|  |  |  |  |  |  | **Lower Bound** | **Upper Bound** |
| LSD | Control | 50ppm | -4.00000 | 2.98142 | .217 | -10.8752 | 2.8752 |
|  |  | 75ppm | -2.00000 | 2.98142 | .521 | -8.8752 | 4.8752 |
|  |  | 100ppm | -12.33333^*^ | 2.98142 | .003 | -19.2085 | -5.4582 |
|  | 50ppm | Control | 4.00000 | 2.98142 | .217 | -2.8752 | 10.8752 |
|  |  | 75ppm | 2.00000 | 2.98142 | .521 | -4.8752 | 8.8752 |
|  |  | 100ppm | -8.33333^*^ | 2.98142 | .023 | -15.2085 | -1.4582 |
|  | 75ppm | Control | 2.00000 | 2.98142 | .521 | -4.8752 | 8.8752 |
|  |  | 50ppm | -2.00000 | 2.98142 | .521 | -8.8752 | 4.8752 |
|  |  | 100ppm | -10.33333^*^ | 2.98142 | .008 | -17.2085 | -3.4582 |
|  | 100ppm | Control | 12.33333^*^ | 2.98142 | .003 | 5.4582 | 19.2085 |
|  |  | 50ppm | 8.33333^*^ | 2.98142 | .023 | 1.4582 | 15.2085 |
|  |  | 75ppm | 10.33333^*^ | 2.98142 | .008 | 3.4582 | 17.2085 |
| Dunnett T3 | Control | 50ppm | -4.00000^*^ | .81650 | .034 | -7.5649 | -.4351 |
|  |  | 75ppm | -2.00000 | .81650 | .262 | -5.5649 | 1.5649 |
|  |  | 100ppm | -12.33333 | 4.13656 | .265 | -42.5827 | 17.9160 |
|  | 50ppm | Control | 4.00000^*^ | .81650 | .034 | .4351 | 7.5649 |
|  |  | 75ppm | 2.00000 | .81650 | .262 | -1.5649 | 5.5649 |
|  |  | 100ppm | -8.33333 | 4.13656 | .472 | -38.5827 | 21.9160 |
|  | 75ppm | Control | 2.00000 | .81650 | .262 | -1.5649 | 5.5649 |
|  |  | 50ppm | -2.00000 | .81650 | .262 | -5.5649 | 1.5649 |
|  |  | 100ppm | -10.33333 | 4.13656 | .350 | -40.5827 | 19.9160 |
|  | 100ppm | Control | 12.33333 | 4.13656 | .265 | -17.9160 | 42.5827 |
|  |  | 50ppm | 8.33333 | 4.13656 | .472 | -21.9160 | 38.5827 |
|  |  | 75ppm | 10.33333 | 4.13656 | .350 | -19.9160 | 40.5827 |
| ***. The mean difference is significant at the 0.05 level.** | | | | | | | |

**Supplementary Table 7.2: Means for groups in homogeneous subsets are displayed of numbers of pods per crop of *Abelmoschus esculantus.***

| **Number of pods per crop** | | | | |
| --- | --- | --- | --- | --- |
| **Duncan^a^** | **Treatment** | **N** | **Subset for alpha = 0.05** | |
|  |  |  | **1** | **2** |
|  | Control | 3 | 4.0000 |  |
|  | 75ppm | 3 | 6.0000 |  |
|  | 50ppm | 3 | 8.0000 |  |
|  | 100ppm | 3 |  | 16.3333 |
|  | Sig. |  | .234 | 1.000 |
| **Means for groups in homogeneous subsets are displayed.** | | | | |
| **a. Uses Harmonic Mean Sample Size = 3.000.** | | | | |

**Supplementary Table 8: Descriptive mean value analysis of weight of pod of *Abelmoschus esculantus* crop variety Bhindi Pusa 5 (DOV 66) at 0.05 significant level through Two-Way Anova analysis.**

| **Descriptives** | | | | | | | | |
| --- | --- | --- | --- | --- | --- | --- | --- | --- |
|  | **N** | **Mean** | **Std. Deviation** | **Std. Error** | **95% Confidence Interval for Mean** | | **Minimum** | **Maximum** |
|  |  |  |  |  | **Lower Bound** | **Upper Bound** |  |  |
| Control | 3 | 10.4300 | .37510 | .21656 | 9.4982 | 11.3618 | 10.00 | 10.69 |
| 50ppm | 3 | 23.1100 | 4.24654 | 2.45174 | 12.5610 | 33.6590 | 20.50 | 28.01 |
| 75ppm | 3 | 26.9667 | 2.60832 | 1.50591 | 20.4872 | 33.4461 | 24.00 | 28.90 |
| 100ppm | 3 | 24.1333 | .32146 | .18559 | 23.3348 | 24.9319 | 23.90 | 24.50 |
| Total | 12 | 21.1600 | 6.97164 | 2.01254 | 16.7304 | 25.5896 | 10.00 | 28.90 |

| **Supplementary Table 8.1: Multiples comparison with dependent variables of weight of pod of *Abelmoschus esculantus* crop variety Bhindi Pusa 5 (DOV 66) at 0.05 significant level.** | | | | | | | |
| --- | --- | --- | --- | --- | --- | --- | --- |
| **Multiple Comparisons** | | | | | | | |
| **Dependent Variable: Pod Weight** | | | | | | | |
|  | **(I) Treatment** | **(J) Treatment** | **Mean Difference (I-J)** | **Std. Error** | **Sig.** | **95% Confidence Interval** | |
|  |  |  |  |  |  | **Lower Bound** | **Upper Bound** |
| LSD | Control | 50ppm | -12.68000^*^ | 2.04452 | .000 | -17.3947 | -7.9653 |
|  |  | 75ppm | -16.53667^*^ | 2.04452 | .000 | -21.2513 | -11.8220 |
|  |  | 100ppm | -13.70333^*^ | 2.04452 | .000 | -18.4180 | -8.9887 |
|  | 50ppm | Control | 12.68000^*^ | 2.04452 | .000 | 7.9653 | 17.3947 |
|  |  | 75ppm | -3.85667 | 2.04452 | .096 | -8.5713 | .8580 |
|  |  | 100ppm | -1.02333 | 2.04452 | .630 | -5.7380 | 3.6913 |
|  | 75ppm | Control | 16.53667^*^ | 2.04452 | .000 | 11.8220 | 21.2513 |
|  |  | 50ppm | 3.85667 | 2.04452 | .096 | -.8580 | 8.5713 |
|  |  | 100ppm | 2.83333 | 2.04452 | .203 | -1.8813 | 7.5480 |
|  | 100ppm | Control | 13.70333^*^ | 2.04452 | .000 | 8.9887 | 18.4180 |
|  |  | 50ppm | 1.02333 | 2.04452 | .630 | -3.6913 | 5.7380 |
|  |  | 75ppm | -2.83333 | 2.04452 | .203 | -7.5480 | 1.8813 |
| Dunnett T3 | Control | 50ppm | -12.68000 | 2.46129 | .103 | -31.1632 | 5.8032 |
|  |  | 75ppm | -16.53667^*^ | 1.52141 | .022 | -27.6429 | -5.4305 |
|  |  | 100ppm | -13.70333^*^ | .28521 | .000 | -14.9643 | -12.4424 |
|  | 50ppm | Control | 12.68000 | 2.46129 | .103 | -5.8032 | 31.1632 |
|  |  | 75ppm | -3.85667 | 2.87729 | .711 | -17.8860 | 10.1727 |
|  |  | 100ppm | -1.02333 | 2.45876 | .996 | -19.5746 | 17.5279 |
|  | 75ppm | Control | 16.53667^*^ | 1.52141 | .022 | 5.4305 | 27.6429 |
|  |  | 50ppm | 3.85667 | 2.87729 | .711 | -10.1727 | 17.8860 |
|  |  | 100ppm | 2.83333 | 1.51731 | .520 | -8.3754 | 14.0420 |
|  | 100ppm | Control | 13.70333^*^ | .28521 | .000 | 12.4424 | 14.9643 |
|  |  | 50ppm | 1.02333 | 2.45876 | .996 | -17.5279 | 19.5746 |
|  |  | 75ppm | -2.83333 | 1.51731 | .520 | -14.0420 | 8.3754 |
| ***. The mean difference is significant at the 0.05 level.** | | | | | | | |

**Supplementary Table 8.2: Means for groups in homogeneous subsets are displayed of pod weight of *Abelmoschus esculantus.***

| **Pod Weight** | | | | |
| --- | --- | --- | --- | --- |
| **Duncan^a^** | Treatment | N | **Subset for alpha = 0.05** | |
|  |  |  | 1 | 2 |
|  | Control | 3 | 10.4300 |  |
|  | 50ppm | 3 |  | 23.1100 |
|  | 100ppm | 3 |  | 24.1333 |
|  | 75ppm | 3 |  | 26.9667 |
|  | Sig. |  | 1.000 | .108 |
| **Means for groups in homogeneous subsets are displayed.** | | | | |
| **a. Uses Harmonic Mean Sample Size = 3.000.** | | | | |

**Supplementary Table 9: Descriptive mean value analysis of seed quantity per pods of *Abelmoschus esculantus* crop variety Bhindi Pusa 5 (DOV 66).**

| **Descriptives** | | | | | | | | | |
| --- | --- | --- | --- | --- | --- | --- | --- | --- | --- |
|  | **N** | **Mean** | **Std. Deviation** | **Std. Error** | **95% Confidence Interval for Mean** | | **Minimum** | **Maximum** |  |
|  |  |  |  |  | **Lower Bound** | **Upper Bound** |  |  |  |
| Control | 3 | 30.3000 | .60828 | .35119 | 28.7890 | 31.8110 | 29.90 | 31.00 |  |
| 50ppm | 3 | 82.3000 | .60828 | .35119 | 80.7890 | 83.8110 | 81.90 | 83.00 |  |
| 75ppm | 3 | 97.3000 | .60828 | .35119 | 95.7890 | 98.8110 | 96.90 | 98.00 |  |
| 100ppm | 3 | 91.3300 | 1.15182 | .66501 | 88.4687 | 94.1913 | 90.00 | 92.00 |  |
| Total | 12 | 75.3075 | 27.71569 | 8.00083 | 57.6978 | 92.9172 | 29.90 | 98.00 |  |

| **Supplementary Table 9.1: Multiple comparisons with dependent variables of seed quantity per pods of *Abelmoschus esculantus* crop variety Bhindi Pusa 5 (DOV 66) at 0.05 significant level.** | | | | | | | |
| --- | --- | --- | --- | --- | --- | --- | --- |
| **Multiple Comparisons** | | | | | | | |
| **Dependent Variable: Seed quantity** | | | | | | | |
|  | **(I) Treatment** | **(J) Treatment** | **Mean Difference (I-J)** | **Std. Error** | **Sig.** | **95% Confidence Interval** | |
|  |  |  |  |  |  | **Lower Bound** | **Upper Bound** |
| LSD | Control | 50ppm | -52.00000^*^ | .63727 | .000 | -53.4696 | -50.5304 |
|  |  | 75ppm | -67.00000^*^ | .63727 | .000 | -68.4696 | -65.5304 |
|  |  | 100ppm | -61.03000^*^ | .63727 | .000 | -62.4996 | -59.5604 |
|  | 50ppm | Control | 52.00000^*^ | .63727 | .000 | 50.5304 | 53.4696 |
|  |  | 75ppm | -15.00000^*^ | .63727 | .000 | -16.4696 | -13.5304 |
|  |  | 100ppm | -9.03000^*^ | .63727 | .000 | -10.4996 | -7.5604 |
|  | 75ppm | Control | 67.00000^*^ | .63727 | .000 | 65.5304 | 68.4696 |
|  |  | 50ppm | 15.00000^*^ | .63727 | .000 | 13.5304 | 16.4696 |
|  |  | 100ppm | 5.97000^*^ | .63727 | .000 | 4.5004 | 7.4396 |
|  | 100ppm | Control | 61.03000^*^ | .63727 | .000 | 59.5604 | 62.4996 |
|  |  | 50ppm | 9.03000^*^ | .63727 | .000 | 7.5604 | 10.4996 |
|  |  | 75ppm | -5.97000^*^ | .63727 | .000 | -7.4396 | -4.5004 |
| Dunnett T3 | Control | 50ppm | -52.00000^*^ | .49666 | .000 | -54.1684 | -49.8316 |
|  |  | 75ppm | -67.00000^*^ | .49666 | .000 | -69.1684 | -64.8316 |
|  |  | 100ppm | -61.03000^*^ | .75204 | .000 | -64.9324 | -57.1276 |
|  | 50ppm | Control | 52.00000^*^ | .49666 | .000 | 49.8316 | 54.1684 |
|  |  | 75ppm | -15.00000^*^ | .49666 | .000 | -17.1684 | -12.8316 |
|  |  | 100ppm | -9.03000^*^ | .75204 | .004 | -12.9324 | -5.1276 |
|  | 75ppm | Control | 67.00000^*^ | .49666 | .000 | 64.8316 | 69.1684 |
|  |  | 50ppm | 15.00000^*^ | .49666 | .000 | 12.8316 | 17.1684 |
|  |  | 100ppm | 5.97000^*^ | .75204 | .015 | 2.0676 | 9.8724 |
|  | 100ppm | Control | 61.03000^*^ | .75204 | .000 | 57.1276 | 64.9324 |
|  |  | 50ppm | 9.03000^*^ | .75204 | .004 | 5.1276 | 12.9324 |
|  |  | 75ppm | -5.97000^*^ | .75204 | .015 | -9.8724 | -2.0676 |
| ***. The mean difference is significant at the 0.05 level.** | | | | | | | |

**Supplementary Table 9.2: Means for groups in homogeneous subsets are displayed of seed quantity per pods of *Abelmoschus esculantus.***

| **Seed quantity** | | | | | | |
| --- | --- | --- | --- | --- | --- | --- |
| **Duncan^a^** | **Treatment** | **N** | **Subset for alpha = 0.05** | | | |
|  |  |  | **1** | **2** | **3** | **4** |
|  | Control | 3 | 30.3000 |  |  |  |
|  | 50ppm | 3 |  | 82.3000 |  |  |
|  | 100ppm | 3 |  |  | 91.3300 |  |
|  | 75ppm | 3 |  |  |  | 97.3000 |
|  | Sig. |  | 1.000 | 1.000 | 1.000 | 1.000 |
| **Means for groups in homogeneous subsets are displayed.** | | | | | | |
| **a. Uses Harmonic Mean Sample Size = 3.000.** | | | | | | |
